# Phosphatidylethanolamine binds to human perilipins *via* a hydrophobic cleft in their 4-helix bundle domain for lipid droplet targeting

**DOI:** 10.1016/j.jbc.2026.113268

**Published:** 2026-06-19

**Authors:** Jiri Stribny, Roger Schneiter

**Affiliations:** Department of Biology, University of Fribourg, Chemin du Musée 10, Fribourg, Switzerland

**Keywords:** amphipathic helices, apolipoprotein E (ApoE), lipid packing defects, neutral lipids, metabolic dysfunction-associated steatotic liver disease (MASLD), nascent LDs, 11-mer repeats, PEMT inhibition, surface tension

## Abstract

Perilipins (PLINs) are a family of proteins that coat the surface of lipid droplets (LDs), the cell's main storage sites for fats, to control their formation, growth, and breakdown. These proteins share a common structure: an N-terminal PAT domain for initial targeting, a central region of repeating helices that insert into the LD surface, and a C-terminal 4-helix bundle for stable anchoring. While the PAT domain binds diacylglycerol to promote LD formation at the endoplasmic reticulum, the conserved 4-helix bundle's lipid-sensing role has remained elusive. Here, we show that this bundle contains a hydrophobic cleft that specifically binds phosphatidylethanolamine (PE), a cone-shaped lipid promoting membrane bending during LD budding, as predicted by AlphaFold3 (alphafoldserver.com) models and confirmed by docking simulations. Binding assays reveal that the isolated bundle strongly attaches to LD-like particles enriched in PE, but mutations closing the cleft block this interaction. In yeast cells, limiting PE reduces PLIN3 localization to LDs, an effect aggravated by cleft mutations but independent of LD size or number. This PE-binding ability is shared by PLIN2, PLIN4, and PLIN5 but missing in PLIN1, matching their structural differences. Overall, our work reveals how the 4-helix bundle lets PLINs detect and adapt to LD surface lipid makeup, explaining their varied cellular roles and opening paths for treatments in fat-storage diseases such as liver steatosis.

Lipid droplets (LDs) are ubiquitous cellular compartments that serve as dynamic stores for neutral lipids, primarily triacylglycerols (TAGs) and sterol esters (SEs). These organelles originate from the endoplasmic reticulum (ER) membrane, where neutral lipids accumulate between leaflets and bud towards the cytosol as mature LDs enveloped by a phospholipid monolayer. LDs maintain energy homeostasis, support membrane biogenesis, and mediate lipid signaling, but their dysregulation underlies human pathologies including obesity, insulin resistance, metabolic dysfunction-associated steatotic liver disease (MASLD), atherosclerosis *via* foam cell formation, and neurodegeneration through inflammatory LD buildup (1, 2). Recent reviews underscore the multifaceted metabolic roles of LDs and therapeutic promise in these disorders (3, 4, 5, 6, 7).

The perilipin (PLIN) family (PLIN1–5) ranks among the most abundant LD surface proteins, forming protective coats that stabilize droplets by lowering surface tension, averting coalescence, and barring lipases from the neutral core (8, 9, 10). PLINs also orchestrate lipid mobilization by interfacing with lipases like adipose triglyceride lipase, hormone-sensitive lipase, and CGI-58, fine-tuning lipolysis under hormonal cues such as catecholamines (11, 12, 13, 14). Expression patterns confer tissue specificity: PLIN1 dominates adipocytes/steroidogenic cells for regulated lipolysis; PLIN2/3 are ubiquitous, peaking in liver/macrophages for basal coating; PLIN4 enriches adipose/muscle; and PLIN5 favors oxidative tissues (heart, brown fat, muscle) for fatty acid oxidation (15, 16). Beyond metabolism, PLIN2/3 have been implicated in cancer progression and viral replication by hijacking LDs (17, 18).

Structurally, PLINs (barring PLIN4) feature a conserved domain architecture: an N-terminal PAT (Perilipin, Adipophilin, TIP47) for LD recognition, a central 11-mer amphipathic helices sensing packing defects from underlying neutral lipids, and a C-terminal 4-helix bundle for LD anchoring and potential protein interactions (10, 19, 20). The PAT domain confers PKA-dependent phosphorylation and translocation from cytosol to LDs, the 11-mer repeats insert hydrophobically into monolayers, and the 4-helix bundle enhances LD affinity (21, 22, 23). The PAT motif further confers selectivity for diacylglycerol (DAG)-enriched ER docking on nascent LDs (5, 24, 25, 26, 27). Resembling apolipoproteins, the 4-helix bundle flexes in lipid environments, akin to apolipoprotein E (ApoE), enabling adaptable lipid remodeling (28, 29). Dysfunctions, *e.g.*, PLIN1 mutations in lipodystrophy or upregulated PLINs in tumors, disrupt lipid flux, and may fuel metabolic cancers (17). Collectively, PLIN domains synergize for targeted diversity, with tension/membrane cues sorting PLINs to LDs of different size and/or composition (10, 30, 31).

PLIN3 emerges as a versatile PLIN, driving LD biogenesis, trafficking, and stress responses including inflammation and viral replication (10, 32, 33). Constitutively expressed unlike restricted PLIN1/5, PLIN3 compensates in knockouts, underscoring its adaptability (34). It coats small nascent LDs, gets displaced by PLIN2 on maturation, and favors high-tension/phospholipid-specific surfaces (31). Prior studies using pendant drop tensiometry illuminated PLIN3 domain specializations in LD interfaces (35, 36). Yet, the functional characterization of the lipid-binding properties of the 4-helix bundle lags those of the PAT and 11-mer repeat domains. Here, we probe its interaction with phospholipids, unveiling a hydrophobic cleft that specifically binds phosphatidylethanolamine (PE) and governs PLIN3 LD recruitment under PE scarcity.

## Results

### The 4-helix bundle domain of PLIN3 binds phosphatidylethanolamine in artificial LDs

**Figure 1:**
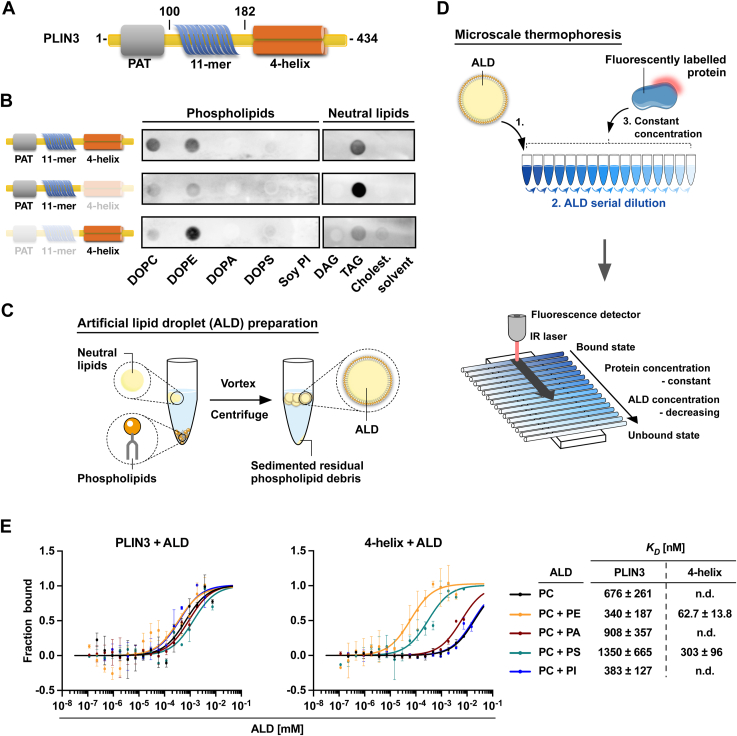
**The 4-helix bundle of PLIN3 shows high affinity binding to ALDs containing PE.***A*, diagram of domain organization of PLIN3 with amino acid positions that delineate individual domains indicated. The PAT domain of PLIN3 (amino acids 1–100, *gray*) is predicted to adopt a triangular shaped structure composed of amphipathic helices. The central 11-mer repeat region (amino acids 101–182, *blue*) contains amphipathic helices that sense lipid-packing defects. The C-terminal 4-helix bundle domain of PLIN3 (amino acids 183–434, *orange*) harbors a central hydrophobic cleft. *B*, protein–lipid overlay assay of full-length PLIN3 and its domains binding to indicated lipids. Lipids immobilized on nitrocellulose membranes were incubated with purified His-tagged proteins, and bound proteins were detected by anti-His immunoblotting. *C*, schematic representation of the preparation of ALDs. Dried phospholipids are hydrated with buffer, mixed with neutral lipids by vortexing, and the resulting ALDs are separated from large lipid particles by centrifugation. *D*, experimental setup of the measurement of the binding affinity of PLIN3 to ALDs by MST. (1) ALDs are added as a binding partner and (2) serially diluted to generate a range of concentrations for binding analysis. (3) A constant concentration of fluorescently labeled PLIN3 is added to each dilution step. The mixtures are loaded into MST capillaries, in which temperature gradient is generated by an infrared laser. Concentration-dependent changes in thermophoretic mobility of the fluorescently labeled protein are detected as variations in fluorescence intensity, which are used to determine a *K*_*D*_ and thus the binding affinity of the protein to ALDs. *E*, binding of full length PLIN3 or that of the 4-helix bundle domain to ALDs composed of PC (DOPC) and spiked (10 mol%) with either PE (DOPE), PA (DOPA), PS (DOPS), or PI (soy PI), measured by MST. The fraction of the fluorescent protein bound to ALDs is plotted against the concentration of ALDs, and the dissociation constant is indicated (mean ± S.D. from three independent experiments). ALD, artificial lipid droplet; MST, microscale thermophoresis; *K*_*D*_, dissociation constant

To investigate the lipid-binding capacity of full-length PLIN3 and its individual domains (Fig. 1*A*), we first performed a protein–lipid overlay assay (Fig. 1*B*). Purified His-tagged full-length PLIN3, the isolated N-terminal PAT–11mer, or the 4-helix bundle domain were incubated with phospholipids and neutral lipids immobilized on nitrocellulose membranes, including dioleoylphosphatidylcholine (DOPC), dioleoylphosphatidylethanolamine (DOPE), dioleoyl phosphatidic acid (DOPA), dioleoylphosphatidylserine (DOPS), soy-derived phosphatidylinositol (soy PI), DAG, TAG, and cholesterol. Full-length PLIN3 bound to DOPC, DOPE and TAG. The PAT–11-mer domain bound primarily to TAG, consistent with previous observations that amphipathic helices within the 11-mer region have high affinity for triacylglycerol (22, 23). The isolated 4-helix bundle domain bound weakly to TAG but, interestingly, displayed a clear preference to DOPE.

To further quantify these interactions in a more physiological context, we examined binding of full-length PLIN3 and its C-terminal 4-helix bundle domain to artificial lipid droplets (ALDs) containing a neutral lipid core composed of triolein:cholesteryl ester (2:1 mol%) using microscale thermophoresis (MST) (Fig. 1, *C* and *D*). MST is an immobilization-free technique that quantifies biomolecular interactions based on the thermophoretic movement of fluorescently labeled molecules in a temperature gradient. Upon ligand binding, the diffusion rate of the labeled protein changes, allowing quantification of binding affinity by plotting fluorescence changes against ligand concentrations (37).

Purified His-tagged human PLIN3 and its isolated C-terminal 4-helix domain were fluorescently labeled and incubated with ALDs of defined phospholipid composition. As a base, ALDs were prepared using DOPC, the major phospholipid of the ER membrane, and supplemented with 10 mol% of either DOPE, DOPA, DOPS, or soy PI.

PLIN3 displayed saturable binding to all the ALDs with similarly affinity, indicating that the full-length PLIN3 did not exhibit a marked lipid preference, consistent with previous binding studies with PLIN2 (38) (Fig. 1*E*). In contrast, the isolated 4-helix bundle did not detectably bind to DOPC containing ALDs, but exhibited strong selective binding to PE-containing ALDs, with a dissociation constant (*K*_*D*_) of 62.7 nM, and to a lesser extent to PS-containing ALDs (*K*_*D*_ of 303 nM), suggesting that the C-terminal 4-helix domain confers phospholipid binding specificity, particularly for PE. This selectivity aligns with recent models of LD surface editing, where phospholipids like PE facilitate LD budding from the ER by promoting negative curvature and with the ability of amphipathic helices to sense such defects (22, 31).

### The 4-helix domain of PLIN3 harbors binding specificity for phosphatidylethanolamine

**Figure 2:**
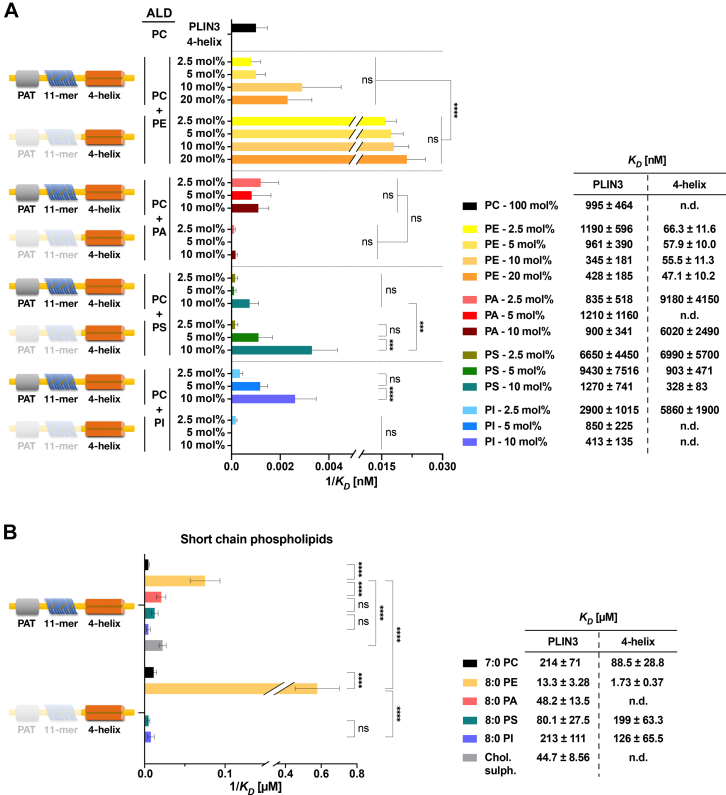
**Probing the binding selectivity of the 4-helix bundle of PLIN3.***A*, binding of full length PLIN3 or that of the 4-helix bundle domain to ALDs composed of PC (DOPC) supplemented with different concentrations of the indicated phospholipids, PE (DOPE), PA (DOPA), PS (DOPS), or PI (SoyPI), measured by MST. *B*, lipid-binding specificity of PLIN3 or that of the 4-helix bundle domain were assessed with short chain 8:0 phospholipids or with cholesterol sulphate (chol. sulph.). *K*_*D*_ are plotted as reciprocal values (mean ± S.D. of three independent measurements). ∗∗∗*p* < 0.001; ∗∗∗∗*p* < 0.0001; ns, not significant (two-way ANOVA with Tukey's *post hoc* test); n.d., not detected. ALD, artificial lipid droplet; MST, microscale thermophoresis; *K*_*D*_, dissociation constant

To characterize the lipid-binding properties of the 4-helix domain of PLIN3 in more detail, we systematically varied the concentration of phospholipids within DOPC-based ALDs and compared the binding properties of full-length PLIN3 to that of the 4-helix bundle domain. Consistent with the previous observations, the 4-helix bundle domain displayed selective binding to PE-containing ALDs across all tested concentrations (2.5–20 mol%), with *K*_*D*_ ranging from 47.1 nM to 66.3 nM. Interestingly, we did not observe a PE-dose dependent change in *K*_*D*_ values, suggesting that binding saturation occurs at low PE levels, as observed before with DAG selectivity binding by the PAT domain of PLIN3 (26, 27). These affinities were at least six-fold stronger than those measured with ALDs composed solely of DOPC or those spiked with other phospholipids (Fig. 2*A* and Table S1).

To further validate this PE binding selectivity of the 4-helix domain, we performed MST binding assays using water-soluble lipid analogs, including short-chain phospholipids (7:0 and 8:0 species) as well as cholesterol sulfate. Among the ligands tested, 8:0 PE emerged as a preferred ligand for both full-length PLIN3 and the 4-helix domain. However, the 4-helix domain alone bound 8:0 PE with a 7.6-fold tighter affinity (*K*_*D*_ of 1.73 μM *versus* 13.3 μM for the full-length protein), reflecting a marked enhancement in binding specificity (Fig. 2*B*). These findings underscore the lipid binding specificity of the C-terminal 4-helix domain for PE, possibly driven by its headgroup charge and conical shape, suggesting that PE’s negative curvature enhances binding (31, 39).

### PLIN3 binds PE-containing ALDs mimicking ER membranes

**Figure 3:**
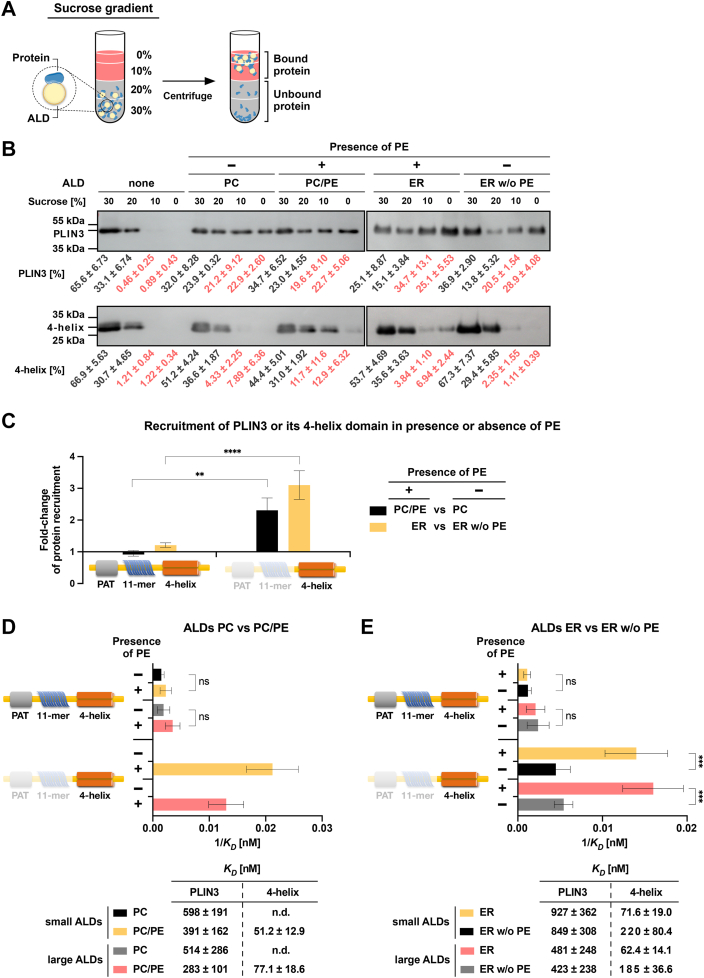
**PE promotes the binding of the 4-helix bundle to ALDs.***A*, schematic representation of the ALD flotation assay used to examine lipid binding specificity of the 4-helix bundle. Full-length PLIN3 or its 4-helix bundle domain was incubated with ALDs of either simple phospholipid composition (DOPC or DOPC/DOPE, containing 20 mol% DOPE), a complex phospholipid composition mimicking that of the ER membrane (DOPC, 53 mol%; DOPE, 23 mol%; DOPS, 8 mol%; DOPA, 5 mol%; soy PI, 11 mol%), or an ER-like composition lacking DOPE (ER w/o PE). All ALDs were prepared using the same phospholipid:triolein:cholesteryl oleate ratio (1:2:1). Protein–ALD mixtures were overlaid with a sucrose gradient and centrifuged. ALD-bound proteins migrated to the upper gradient fractions. *B*, analysis of protein distribution in different gradient fractions. Proteins were separated by SDS-PAGE and probed by an anti-His antibody. Band intensities were quantified and are presented as percentage of total signal per sample (mean ± S.D. from three independent experiments). *C*, quantification of ALD recruitment of full-length PLIN3 and the 4-helix bundle domain in the presence or absence of PE, as assayed by ALD flotation. Fold-change in recruitment was calculated as the ratio of protein bound to PE-containing ALDs (DOPC/DOPE or ER-mimicking composition) relative to ALDs lacking PE (DOPC alone or ER w/o PE, respectively). ∗∗*p* < 0.01; ∗∗∗∗*p* < 0.0001; (two-way ANOVA with Bonferroni's *post hoc* test). *D* and *E*, MST analysis of full-length PLIN3 and the 4-helix bundle domain binding to ALDs lacking or containing PE: DOPC alone (PE–) or DOPC/DOPE (PE+) for simple ALDs (*panel D*), and ER-mimicking composition (PE+) or ER w/o PE (PE–) for complex ALDs (*panel E*). Binding was assessed using both standard ALDs (140–180 nm) and large ALDs (320–380 nm). Dissociation constants are plotted as reciprocal values (mean ± S.D. of three independent measurements). ∗∗∗*p* < 0.001; ns, not significant (two-way ANOVA with Bonferroni's *post hoc* test); n.d., not detected. ALD, artificial lipid droplet; MST, microscale thermophoresis; ER, endoplasmic reticulum

To corroborate these MST-based results, we examined the recruitment of PLIN3 and its C-terminal 4-helix bundle domain to PE-containing *versus* PE-deficient ALDs using a flotation assay (Fig. 3*A*). Full-length PLIN3 or the isolated 4-helix domain were incubated with ALDs of either simple phospholipid composition (PC or PC/PE) or a more complex phospholipid mixture mimicking that of the ER membrane (DOPC/DOPE/DOPS/DOPA/SoyPI; 53/23/8/5/11 mol%, hereafter termed ER-ALD). An ER-like phospholipid mixture lacking PE (referred to as ER w/o PE) was used as a PE-deficient control. Following incubation, protein-bound ALDs were separated by sucrose gradient flotation and analyzed by Western blotting (Fig. 3*B*).

As expected, full-length PLIN3 showed stronger overall recruitment to ALDs than the isolated 4-helix bundle domain, likely due to the presence of additional lipid-binding domains, particularly the N-terminal PAT and 11-mer repeat domains. However, when recruitment was expressed as a ratio between PE-containing and PE-lacking ALDs, the 4-helix bundle domain showed a clear PE preference. It bound >2-fold more strongly to PC/PE-ALDs compared to PC-ALDs alone, and >3-fold more to ER-ALDs than to ER w/o PE-ALDs (Fig. 3, *B* and *C*). In contrast, full-length PLIN3 was recovered at similar levels from all types of ALDs, regardless of their PE content.

To further validate this PE selectivity, we performed MST experiments using the same ALD compositions as in the flotation assay. Consistent with the flotation results, the 4-helix bundle domain displayed selective binding to PE-containing ALDs. Binding was detected for PC/PE ALDs (*K*_*D*_ = 51.2 nM), while no binding was observed for PC-ALDs. Similarly, the 4-helix domain bound ER-ALDs with higher affinity than ER w/o PE (*K*_*D*_ = 71.6 nM *versus* 220 nM, respectively), representing a ∼3-fold difference (Figs. 2*A*, and 3, *D* and *E*). To assess whether the high curvature of the small ALDs (∼140–180 nm diameter) contributed to the observed PE selectivity, we generated larger ALDs (∼320–380 nm diameter) by reducing the phospholipid content during ALD preparation and repeated the MST analysis. The 4-helix bundle domain retained selective binding to PE-containing large ALDs, binding PC/PE-ALDs with a *K*_*D*_ of 77.1 nM while no binding was detected for large PC-ALDs. Likewise, the 4-helix domain bound large ER-ALDs with substantially higher affinity than large ER w/o PE-ALDs (*K*_*D*_ = 62.4 nM *versus* 185 nM, respectively) (Fig. 3, *D* and *E*). Together, these results confirm PE’s role in enhancing LD recruitment of PLIN3 *via* the 4-helix bundle domain and indicate that this preferential interaction is largely independent of ALD size and membrane curvature within the tested range.

### Structural modeling reveals a hydrophobic cleft in the 4-helix bundle for PE docking

**Figure 4:**
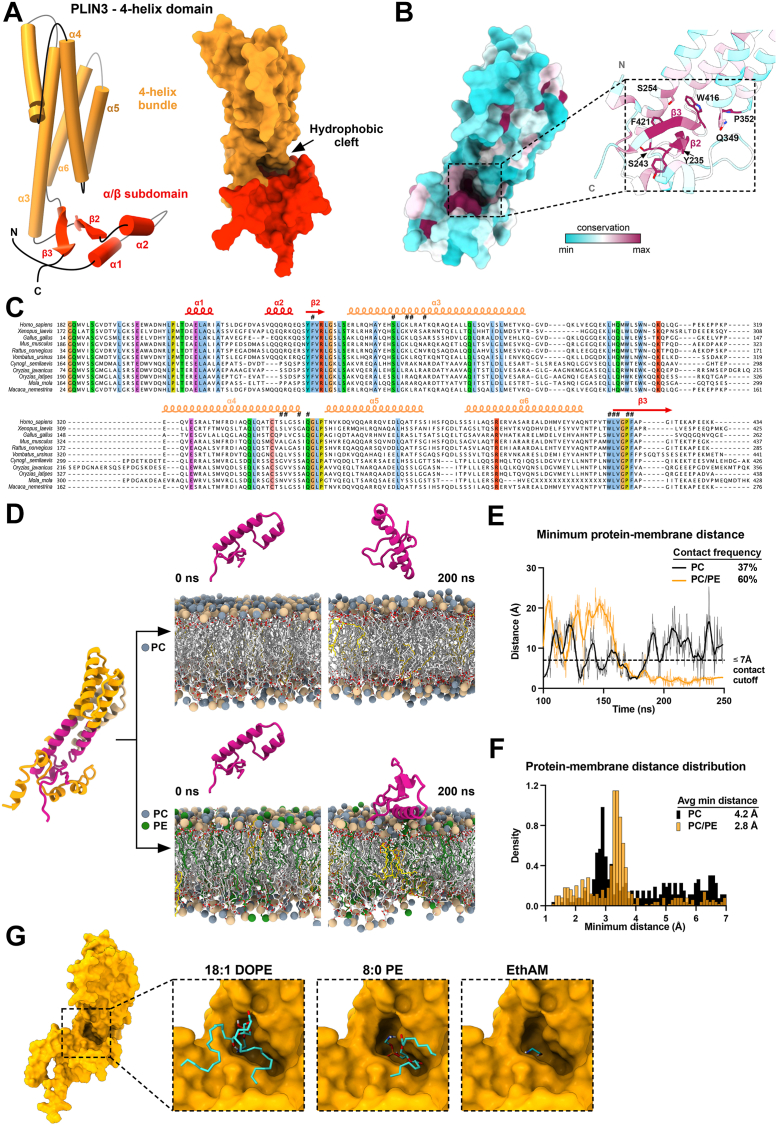
**Structural analysis of the conserved 4-helix bundle domain of PLIN3.***A*, cartoon of the structure of the C-terminal 4-helix domain of PLIN3 as predicted by AlphaFold, showing an elongated four-helix bundle (*orange*) and a compact α/β-subdomain (*red*). *Left-hand side*, simplified secondary structure depiction, with helices shown as cylinders and β-sheets as *arrows*. *Right-hand side*, surface representation highlighting a central hydrophobic cleft between the two subdomains indicated in *orange* and *red*. *B*, surface view of the 4-helix domain colored by sequence conservation, as calculated by the ConSurf server from a multiple sequence alignment of 300 orthologs. The conservation scale ranges from *cyan* (low conservation) to maroon (high conservation). Inset, detailed view of the central hydrophobic cleft, revealing a cluster of highly conserved residues. *C*, multiple sequence alignment of the 4-helix domain of PLIN3 orthologs obtained from the OMA database (orthologous matrix; (40)). The sequences of PLIN3 (OMA Group 1,365,829; Fingerprint WKEKQAG) were downloaded, aligned with MAFFT (41), and curated in Jalview (42) to highlight conserved residues within the 4-helix domain. Secondary structure elements are displayed above the alignment and color-coded as in panel A. Residues forming the central cleft are indicated by #. *D*, all-atom MD simulations of the conserved cleft region in membrane bilayers. The full predicted C-terminal 4-helix domain is shown on the *left*, with the three conserved segments used for the simulation (residues 233–265, 337–349 and 412–429) highlighted in *violet*. These segments were extracted and connected by short flexible GGG linkers. *Top row*: PC bilayer; *bottom row*: PC:PE (80:20) bilayer. *Left* panels show the initial system setup at 0 ns (protein positioned 20 Å above the membrane); *right* panels show representative snapshots after 200 ns of simulation. PC lipids are shown in *gray* with the quaternary amine nitrogen as a *gray sphere*; PE lipids are shown in *green* with the primary amine nitrogen as a green sphere; all lipid phosphorus atoms are drawn as tan-colored spheres; TAG (10%) lipids are shown in *yellow*. *E*, time trace of the minimum protein–membrane distance during the equilibrated phase (100–250 ns). The *dashed line* indicates the protein-membrane contact cutoff (≤7 Å). Raw data are shown as thin lines; bold lines represent Savitzky–Golay smoothed data (window length = 51, polynomial order = 2) for improved visual clarity. *F*, probability density distribution of the minimum protein–membrane distances in contact frames (min distance ≤ 7 Å). *G*, docking of DOPE, short chain 8:0 PE, and ethanolamine (EthAM) into the central hydrophobic cleft within the 4-helix bundle domain of PLIN3 as predicted by AutoDock Vina (43). All ligands were predicted to bind within the central hydrophobic cleft of the 4-helix domain, with the ethanolamine or glycerol ethanolamine moiety oriented towards the cleft interior.

Since there is no experimentally determined structure of the C-terminal domain of human PLIN3 available, we used AlphaFold3 to predict its structure. As previously described for the mouse ortholog (28), the C-terminal domain adopts a characteristic L-shaped fold consisting of a four-helix bundle (α3–α6), an additional perpendicular helix between α3 and α4, and a compact α/β subdomain (Fig. 4*A*). These structural elements together form a deep hydrophobic cleft located at the interface of the two subdomains. This cleft features a deep pocket surrounded by hydrophobic surfaces that might accommodate acyl chains of lipids like PE, promoting insertion into PE-enriched interfaces on the surface of LDs. The residues lining this central cleft are highly conserved among PLIN family members, as revealed by multiple sequence alignment and conservation mapping (Figs. 4, *B* and *C* and 7*B*).

To investigate whether this conserved cleft can preferentially engage PE-containing membranes, we performed all-atom molecular dynamics (MD) simulations of a minimal model system comprising the conserved structural elements around the cleft (residues 233–265, 337–349, and 412–429) (Fig. 4*D*). The fragments were connected by short flexible linkers (glycylglycylglycine [GGG]) introduced during modelling to maintain their relative spatial arrangement. This minimal model was chosen to directly test the behavior of the conserved cleft and avoid potential nonspecific interactions from the full C-terminal region. The protein was initially positioned at a distance of 20 Å from lipid bilayers composed of either pure PC or a PC/PE mixture. In PC/PE membranes, the protein exhibited significantly higher contact frequency and closer average minimum distance to the lipid headgroups compared to pure PC membrane system (60% *versus* 37% contact occupancy at ≤ 7 Å cutoff, and 2.8 Å *versus* 4.2 Å average minimum distance in bound frames) (Fig. 4, *E* and *F*). These results support the hypothesis that the conserved cleft is capable of preferentially interacting with PE-enriched interfaces, likely due to better accommodation of PE conical headgroup geometry and enhanced anchoring by conserved hydrophobic residues.

To further probe the molecular basis of this PE preference, we assessed potential binding sites for PE lipids on the full C-terminal domain. *In silico* ligand docking positioned both PE ligands (18:1 PE and 8:0 PE) and ethanolamine within this conserved cleft at the α3/α4-β3 interface (Fig. 4*G*). Complementary analyses using the protein–ligand interaction profiler and LigPlot confirmed multiple noncovalent interactions, including hydrogen bonds and hydrophobic contacts (44) (Fig. S1*A*). These interactions primarily involved hydrogen bonding by S343, S346 and S347 and van der Waals contacts with hydrophobic residues, including the highly conserved F236 and W416 (Fig. S1, *A* and *B*). This binding mode is reminiscent of lipid pockets in apolipoproteins like ApoE, which exhibits conformational heterogeneity upon lipid association and supports PE's conical geometry fitting into curved clefts to stabilize membrane-protein interfaces (29, 45).

### Cleft mutations disrupt PE binding *in vitro*

**Figure 5:**
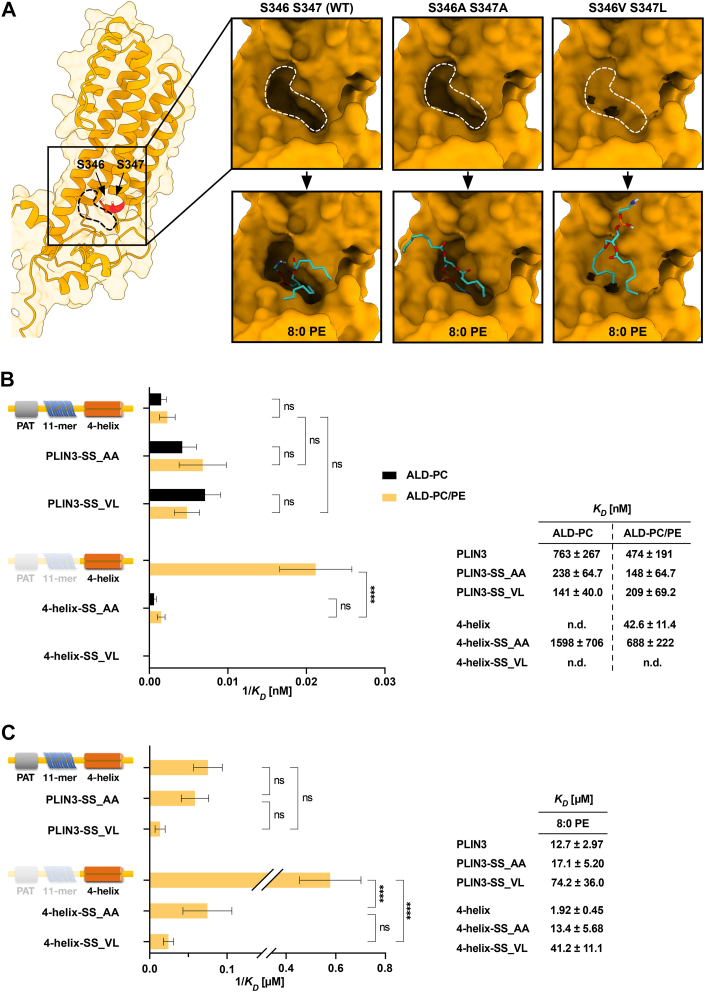
**Cleft accessibility and PE recognition by the 4-helix domain.***A*, cartoon representation of the 4-helix domain of PLIN3 highlighting the rim of the central hydrophobic cleft (*dashed line*). Two mutant variants were modeled: i) the S346A S347A double mutant retained an open cleft like the WT structure, whereas ii) the S346V S347L double mutant exhibited cleft closure due to the presence of bulkier residues, valine and leucine instead of serine. Docking simulations with 8:0 PE were performed for each variant (*boxes below each structure*). The ligand docked within the cleft interior in the WT and S346A S347A models, whereas it was shifted to binding to an external site in the S346V S347L mutant. *B* and *C*, binding of full length PLIN3 or the 4-helix domain, including their mutant variants to ALDs composed of PC (DOPC) alone or spiked with 20 mol% PE (DOPC/DOPE) (*panel B*), or to short chain 8:0 PE (*panel C*), was determined by MST. Dissociation constants are plotted as reciprocal values (mean ± S.D. of three independent measurements). ∗∗∗∗*p* < 0.0001; ns, not significant (two-way ANOVA in *B*; one-way ANOVA in *C*; Tukey's *post hoc* test); n.d., not detected. ALD, artificial lipid droplet; MST, microscale thermophoresis

To further evaluate the potential function of the conserved deep cleft in the C-terminal 4-helix domain of PLIN3 in PE binding, we performed *in silico* mutational modeling of several residues lining the cleft (Fig. S1). Among the modeled variants, two serine residues located at the cleft rim (S346 and S347) emerged as likely influencing cleft accessibility. These residues were therefore selected for further structural and functional analysis (Fig. 5*A*). Substitution of both serine residues with alanine (S346A S347A) was predicted to preserve the open conformation of the cleft, similar to the WT structure. In contrast, replacement with bulkier hydrophobic residues (valine and leucine; S346V S347L) resulted in a closure of the cleft due to increased steric hindrance. Docking simulations with 8:0 PE reflected these predicted changes: the ligand remained docked within the cleft in the WT and alanine variants but was displaced to an external surface site in the S346V S347L mutant.

To test the functional implications of these mutations, we carried out MST binding assays with full-length PLIN3 and the 4-helix bundle domain, as well as their respective mutant versions. Full-length PLIN3 and its cleft mutants bound to all tested ALD compositions with similar affinities, with statistically insignificant differences, isolating the bundle's role in PE selectivity (Fig. 5*B*). In contrast, the 4-helix bundle mutants showed markedly reduced (S346A S347A) or abolished (S346V S347L) binding to PE-containing ALDs, confirming the cleft's functional importance.

A similar trend was observed when using soluble 8:0 PE as ligand (Fig. 5*C*). Full-length PLIN3 and its cleft mutants showed a decreasing trend in binding affinity, though these differences were not statistically significant. However, for the 4-helix domain, binding to 8:0 PE was reduced ∼7-fold in the S346A S347A mutant (*K*_*D*_ of 13.4 μM), and further ∼21-fold in the S346V S347L variant (*K*_*D*_ of 41.2 μM), confirming a functional role of this cleft region in lipid binding. These cleft mutations did not appear to affect the overall structure of the 4-helix bundle domain as indicated by CD measurements, which showed characteristic α-helical signatures with minimal deviations in mutants (Fig. S2). Overall, these data indicate that hydrophobic cleft that is formed between the four-helix bundle (α3–α6), and the α/β subdomain within the 4-helix bundle domain of PLIN3 is important for binding PE.

### PE depletion impairs PLIN3 recruitment to LDs *in vivo*, and this defect is exacerbated by cleft mutations

To investigate whether cellular PE levels influence PLIN3 localization to LDs *in vivo*, we employed *Saccharomyces cerevisiae* as a heterologous system. In yeast LDs, PE constitutes the third most abundant phospholipid (∼20.0 mol%), after phosphatidylcholine and phosphatidylinositol (46). We monitored GFP-tagged PLIN3 in WT and phosphatidylserine decarboxylase *(psd)1Δ psd2Δ* cells, the latter lacking the two phosphatidylserine decarboxylases essential for PE synthesis and exhibiting strongly reduced PE levels upon ethanolamine withdrawal.

**Figure 6:**
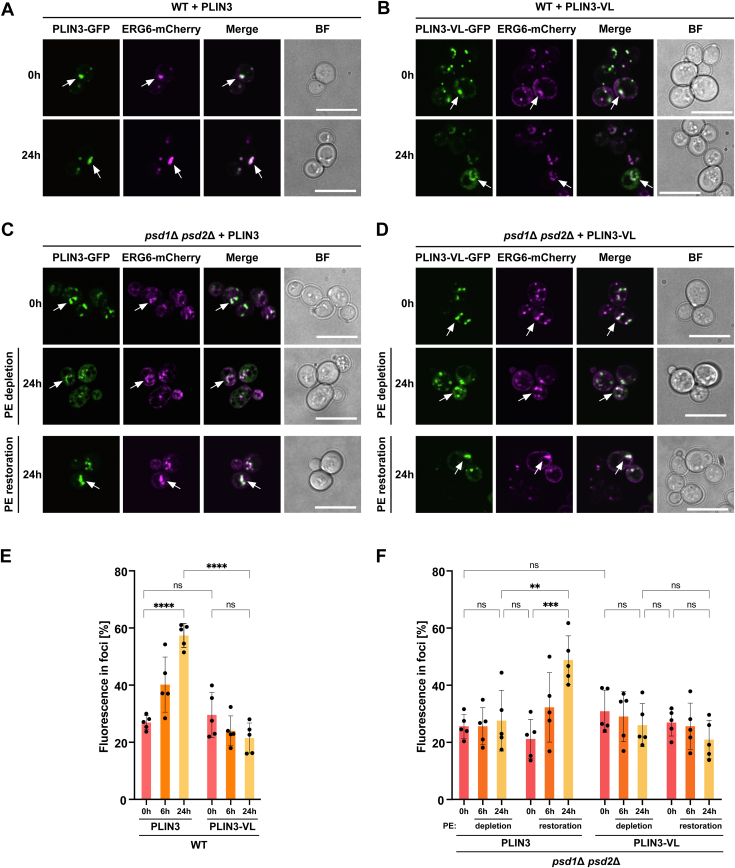
**Cellular PE levels affect recruitment of PLIN3 to LDs in *S. cerevisiae.****A* and *D*, representative images of WT and *psd1Δ psd2Δ* double mutant cells expressing GFP-tagged PLIN3 (*panels A* and *C*) or the PLIN3-S346V S347L (PLIN3-VL) mutant version (*panels B* and *D*), along with genomically tagged Erg6-mCherry as an LD marker. Cells were grown in ethanolamine-supplemented medium and then shifted to ethanolamine-free medium (Time 0 h), and protein localization was monitored over a 24 h period. Under these conditions, WT cells maintain PE levels through endogenous Psd activity, whereas *psd1Δ psd2Δ* cells undergo PE depletion. *psd1Δ psd2Δ* cells (*C* and *D*) were analyzed under PE depletion (EthAM-free medium) and subsequently transferred back to ethanolamine-supplemented medium to examine LD localization of PLIN3 or PLIN3-VL following restoration of PE levels. For clarity, only the 0 h and 24 h timepoints are shown. Arrows indicate the colocalization of the fluorescently labeled proteins. Scale bars represent 10 μm. *E* and *F*, quantification of the fluorescence intensity of the GFP-tagged protein in colocalizing puncta (foci) in WT (*panel E*) and *psd1Δ psd2Δ* cells (*panel F*), expressed as the percentage of total cellular fluorescence at the indicated timepoints. PE depletion, PE-limiting conditions (ethanolamine-free medium); PE restoration (ethanolamine re-supplemented medium). n > 60 cells/strain. Data represent mean ± S.D. of five independent experiments. ∗∗*p* < 0.01; ∗∗∗*p* < 0.001; ∗∗∗∗*p* < 0.0001; ns, not significant (one-way ANOVA with Tukey's *post hoc* test). LD, lipid droplet

Cells were shifted from ethanolamine-supplemented to ethanolamine-free medium at the start of the time course (0 h). In WT cells, where PE levels remain stable independently of external ethanolamine, GFP-PLIN3 showed partial LD colocalization with the marker Erg6-mCherry (∼30% overlap) at 0 h. This association increased progressively to ∼60% after 24 h (Fig. 6, *A* and *E*), consistent with time-dependent recruitment to maturing LDs (10).

In contrast, the cleft mutant GFP-PLIN3-S346V S347L displayed impaired targeting in WT cells, with LD colocalization remaining at ∼30% throughout the 24 h period (Fig. 6, *B* and *E*). This indicates that an intact hydrophobic cleft is required for efficient progressive recruitment.

In *psd1Δ psd2Δ* cells, PE levels drop from ∼24 mol% to ∼7.5 mol% upon ethanolamine withdrawal (47, 48). Under these conditions, WT GFP-PLIN3 failed to undergo progressive LD recruitment and remained at ∼30% colocalization after 24 h (Fig. 6, *C*, *D*, and *F*), phenocopying the cleft mutant in WT cells. Resupplementation with ethanolamine restored LD localization of WT PLIN3 to ∼55%, comparable to WT levels. However, the S346V S347L mutant remained impaired (∼30% colocalization), demonstrating that cleft integrity is essential for PE-dependent rescue (Fig. 6, *D* and *F*).

Importantly, LD number and size were comparable across strains and conditions (Fig. 6, *E* and *F*; quantified in >200 LDs per condition). Total protein abundance and stability of both GFP-PLIN3 and the mutant were equivalent across all conditions and time points, as confirmed by Western blotting (Fig. S3*K*) and cycloheximide chase experiments (Fig. S3). These controls rule out expression or turnover differences as confounding factors.

Collectively, these results demonstrate that sufficient PE availability and an intact C-terminal 4-helix bundle cleft are both required for efficient PLIN3 recruitment to LDs *in vivo*. Disruption of either PE levels or cleft accessibility impairs progressive LD targeting, supporting a direct role for PE sensing by the conserved hydrophobic cleft in LD protein sorting.

### PE binding is conserved in the 4-helix bundle domains of PLIN2, PLIN4 and PLIN5 but divergent in PLIN1

**Figure 7:**
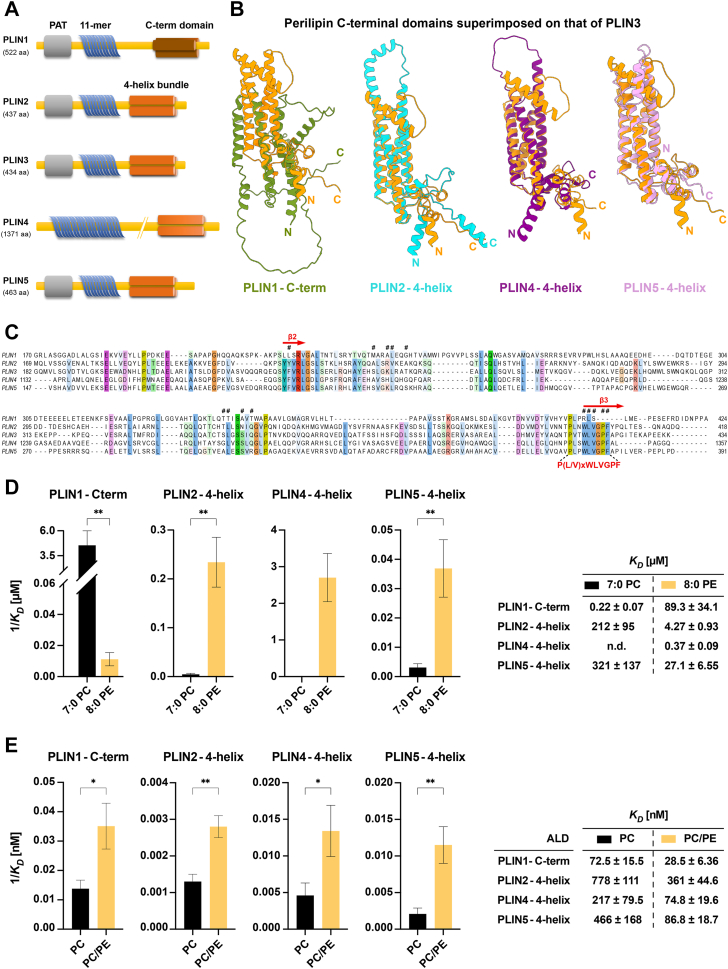
**PE binding by the 4-helix domain is conserved in PLIN2, PLIN4, and PLIN5.***A*, comparative domain architecture of human perilipin family members (PLIN1–PLIN5) showing conserved and divergent structural features: the N-terminal PAT domain (*gray*), the central region containing 11-mer amphipathic helices (*blue*), and the C-terminal 4-helix bundle domain (*orange*). The C-terminal domain adopts a 4-helix bundle fold in PLIN2–PLIN5, whereas in PLIN1 this region contains hydrophobic segments that may form integral and peripheral membrane associantions. *B*, superimposition of the predicted structures of the C-terminal 4-helix domain of PLIN3 with corresponding domains of other PLIN family members, highlighting similar topology among PLIN2, PLIN3, PLIN4, and PLIN5, and a distinct fold in PLIN1. Structures were predicted using AlphaFold3 and aligned to the PLIN3 4-helix domain using the MatchMaker tool in ChimeraX (49). *C*, sequence alignment of the C-terminal domains of PLIN1-PLIN5 confirming the structural divergence shown in *panel A* and *B*. Secondary structure elements (β2 and β3) and residues forming the hydrophobic cleft (indicated by #) are annotated according to the PLIN3 sequence and serve as positional references for other PLIN family members. *D* and *E*, binding of the C-terminal domains of PLIN1, PLIN2, PLIN4, or PLIN5, to short chain phospholipids (*panel D*) or ALDs composed of PC (DOPC) only or spiked with 20 mol% PE (DOPC/DOPE) (*panel E*). Dissociation constants derived from MST measurements are plotted as reciprocal values (mean ± S.D. of three independent measurements). ∗*p* < 0.05; ∗∗*p* < 0.01; (unpaired *t* test, two-tailed); n.d., not detected. ALD, artificial lipid droplet; MST, microscale thermophoresis.

To assess whether the structural features identified in the C-terminal 4-helix bundle domain of PLIN3 are conserved across other members of the perilipin protein family, we compared the predicted structures of the corresponding C-terminal regions of human PLIN1, PLIN2, PLIN4 and PLIN5 (Fig. 7, *A* and *B*). The modeled structures were aligned with the PLIN3 4-helix bundle domain using the structural alignment tool MatchMaker in ChimeraX (49). This analysis revealed that the overall fold and topology of PLIN2, PLIN3, PLIN4, and PLIN5 were highly similar, each adopting a compact arrangement of an elongated four-helix bundle. In contrast, the predicted structure of PLIN1 exhibited a notably different conformation with long unstructured C-terminal tail, lacking the characteristic organization observed in the other paralogs. Sequence alignment further supported these structural differences, highlighting a highly conserved motif P(L/V)xWLVGPF that is embedded in the 4-helix bundle and forms part of the cleft's hydrophobic lining. These findings suggest that the structural motif of the 4-helix bundle and its hydrophobic cleft may be a conserved feature among a subset of PLIN proteins, potentially reflecting a shared mechanism of membrane interaction.

To explore whether these structural differences translate into functional divergence, we next examined the lipid-binding properties of the PLIN1, PLIN2, PLIN4, and PLIN5 C-terminal domains using MST. When tested with short-chain soluble phospholipids, PLIN1 displayed strong interaction with 7:0 PC (*K*_*D*_ of 0.22 μM), whereas PLIN2, PLIN4, and PLIN5 preferentially bound to 8:0 PE (*K*_*D*_ of 4.27 μM, 0.37 μM, and 27.1 μM, respectively), consistent with the lipid binding preference observed for PLIN3 (Fig. 7*D*).

We then measured the binding to ALDs composed of either PC alone or PC/PE mixtures (Fig. 7*E*). Interestingly, all the C-terminal domains showed increased affinity for PE-containing ALDs compared to PC-ALDs. However, PLIN1 displayed considerably stronger overall binding, with affinities approximately 3–10-fold higher than those observed for the C-terminal domains of PLIN2, PLIN4, and PLIN5. The comparatively stronger interaction of PLIN1 may be attributed to additional structural features in its C-terminus, possibly including integral and peripheral membrane segments, as recently proposed (50). Overall, these findings support a conserved role of the 4-helix domain in membrane interaction, with lipid selectivity and binding strength varying among PLIN family proteins.

## Discussion

In this study, we show that the conserved C-terminal 4-helix bundle domain of human PLIN3 binds PE, a key phospholipid in LD biogenesis (Fig. 1). Using microscale thermophoresis, we demonstrate that the isolated 4-helix bundle exhibits nanomolar affinity (*K*_*D*_ of 62.7 nM) for ALDs spiked with just 10 mol% PE, with saturation occurring at low PE concentrations (2.5–20 mol%) and outperforming other phospholipids such as phosphatidylserine (*K*_*D*_ of 303 nM) (Figs. 1, and 2). This selectivity was not apparent in the full-length protein, which bound ALDs nonspecifically, highlighting the bundle’s specialized contribution to overall LD targeting. Flotation assays with ER-mimicking phospholipid compositions further confirmed a >3-fold recruitment preference for PE-containing membranes, consistent with PE’s established role in promoting negative membrane curvature during LD budding from the ER (51) (Fig. 3).

Previous structural analysis of mouse PLIN3 identified a hydrophobic cleft (∼13 × 18 × 10 Å) at the interface between the bundle and α/β subdomain (28). Our data indicate that this cleft is highly conserved across most PLIN family members, with the notable exception of PLIN1. Docking simulations suggest that conserved hydrophobic residues within the β2 and β3 strands engage the phospholipid acyl chains *via* van der Waals interactions, while polar residues at the cleft entry (S254, K257, T261, S343, S346, S347, Q349) form hydrogen bonds with the ethanolamine headgroup (28) (Figs. 4, 5, and S1*B*). This binding mode is well-suited to PE’s conical geometry, potentially facilitating insertion into curved interfaces and packing defects that arise during nascent LD biogenesis. The cleft contains a conserved P(L/V)xWLVGPF motif in the β3 strand that is absent in PLIN1 and contributes to phospholipid selectivity. The tryptophan and terminal phenylalanine support π-π stacking and van der Waals contacts with the PE ligand, while the proline initiates a turn that confers flexibility (52) (Figs. 7, and S1). Consistent with this model, mutations at the cleft rim (S346A S347A) reduced PE specificity ∼7–16-fold, whereas the bulkier S346V S347L substitutions abolished binding through steric occlusion, without affecting the overall α-helical fold as confirmed by circular dichroism (Fig. S2). These results suggest an allosteric gating mechanism in which cleft accessibility modulates PE sensing, reminiscent of lipid-responsive conformational changes in ApoE (29). The 4-helix bundles of PLIN2–5 are structurally analogous to the N-terminal domain of ApoE and were previously implicated in liposome fragmentation into discoidal structures, yet their lipid specificity remained unclear (28, 33).

These findings extend the current model of PLIN domain specialization. The N-terminal PAT domain preferentially binds DAG at ER sites to initiate nascent LD formation (25–27), while the central 11-mer amphipathic helices sense lipid packing defects to stabilize the LD monolayer (22, 23). Here, we demonstrate that the C-terminal 4-helix bundle domain provides an additional layer of specificity by directly sensing phosphatidylethanolamine (PE) *via* its conserved hydrophobic cleft.

The substantially higher affinity of PLIN3 for PE-containing ALDs (KD ≈ 63 nM) compared with soluble short-chain PE (KD ≈ 1.7 μM) indicates that full acyl-chain embedding into the neutral lipid core strongly stabilizes anchoring. This behavior parallels apolipoprotein-mediated remodeling of lipoproteins (30) (Figs. 2, and 3). PE dependence appears biologically meaningful. Recent studies have identified spatially restricted PE pools at LDs generated by localized PSD activity. In mammalian cells, an alternatively spliced PISD isoform localizes to LDs (53), while in yeast Psd1 exhibits dual mitochondrial/ER localization and generates distinct PE pools near a subset of LDs (54, 55).

Consistent with this framework, genetic depletion of PE in *psd1Δ psd2Δ* yeast (reducing cellular PE from ∼24 mol% to ∼7.5 mol%) strongly impaired progressive recruitment of GFP-PLIN3 to Erg6-mCherry-marked LDs, with colocalization stalling at ∼30% over 24 h. This phenotype was phenocopied by the cleft mutant (S346V S347L) in WT cells and was not rescued by ethanolamine resupplementation in the mutant background (Fig. 6). Protein abundance and LD morphology remained comparable across conditions, confirming that the observed defects reflect specific impairments in PE-cleft–dependent targeting (56) (Fig. S3).

PE binding *via* the 4-helix bundle is conserved in PLIN2, PLIN4, and PLIN5, which share key cleft residues (including the P(L/V)xWLVGPF motif) and display 3- to 10-fold preferences for PE (Fig. 7). In contrast, PLIN1 lacks a structured cleft and instead relies on an unstructured C-terminus with transmembrane segments for ER integration (50). Functionally, the PLIN family can be divided into two groups: PLIN2/3/5, which preferentially coat nascent or metabolically dynamic LDs in a curvature- and composition-sensitive manner, and PLIN1, which primarily regulates TAG mobilization in adipocytes through recruitment of lipases (10, 30, 57).

The three major domains of PLIN3 appear to function cooperatively during LD biogenesis. The N-terminal PAT domain initiates ER targeting by binding DAG at sites of nascent LD formation. The central 11-mer amphipathic helices then insert into lipid packing defects in the monolayer, while the C-terminal 4-helix bundle provides additional stabilization through specific PE recognition *via* its hydrophobic cleft. Together, these interactions enable the 4-helix bundle to act as a curvature- and composition-sensing checkpoint that stabilizes nascent LDs emerging from PE-enriched ER protrusions, thereby preventing premature coalescence (3, 6).

This mechanism likely integrates with local phospholipid metabolism. Given that Psd1 in yeast and PISD in mammals can concentrate near forming LDs (53, 54, 55), we hypothesize that local PE enrichment serves as a regulatory cue for protein recruitment. In this model, high PE levels on nascent, rapidly expanding droplets promote PLIN3 association, whereas declining local PSD-dependent PE production on mature droplets reduces PE density, potentially triggering PLIN3 dissociation and its replacement by other perilipins such as PLIN2. This is consistent with reports that C-terminal domains largely determine the relative LD binding affinity and exchange dynamics of different PLINs, with PLIN3 displaying particularly rapid kinetics (24). Such PE-dependent regulation would allow cells to coordinate the temporal succession of LD coat proteins according to droplet growth and metabolic state. Additionally, PE (typically 10–25 mol% on LDs) enhances binding by exposing hydrophobic voids, as observed under conditions that elevate LD PE content (58, 59). This evolutionary partitioning likely underlies functional specialization within the PLIN family: PLIN2/3/5 for early, curvature-driven stabilization of nascent LDs *versus* PLIN1’s primary role in regulated lipolysis.

These observations have implications for metabolic diseases. In MASLD, ethanol inhibits phosphatidylethanolamine N-methyltransferase, lowering PC:PE ratios and enhancing PLIN2/3 LD association, which promotes steatosis, fibrosis, and impaired VLDL secretion (60, 61, 62, 63, 64, 65). Downregulation or deletion of PLIN2 protects against alcohol- and diet-induced hepatic lipid accumulation by enhancing lipophagy and reducing ceramide levels (66, 67, 68), while in the CNS, PLIN3-PE interactions may contribute to microglial LD accumulation in neurodegeneration (2). Dysregulated PLIN3 contributes to MASLD, obesity, and cancer by altering LD dynamics, where PE binding may help PLIN3 mitigate phospholipid scarcity during rapid LD growth. Therapeutically, cleft-targeted modulators could enhance LD turnover in liver disease, redirect lipid metabolism in cancer, or improve lipid handling in steatosis and lipodystrophies (17, 30, 58, 69, 70, 71, 72).

While our biophysical and yeast heterologous models provide compelling mechanistic insight, limitations remain. Reliance on predicted structures limits atomic precision, and heterologous expression may not fully recapitulate mammalian-specific regulators such as PKA phosphorylation or CGI-58 interactions. Future studies should include cryo-EM of PLIN3-LD complexes, CRISPR-based introduction of cleft mutants in hepatocyte models to evaluate effects on lipolysis and disease phenotypes, and exploration of links to PE scramblases in LD biogenesis (10). Integrating these findings with LD-mitochondria coupling (*e.g.*, *via* PLIN5) may further illuminate broader roles in metabolic regulation. Collectively, our results position the 4-helix bundle as a tunable sensor that contributes to LD homeostasis with broad relevance to lipid-related pathologies.

## Experimental procedures

### Materials

Unless otherwise noted, lipids used in this study were purchased from Avanti Polar Lipids. For direct binding assays with PLIN3 or its 4-helix bundle domain, soluble short-chain lipids were used: 1,2-diheptanoyl-sn-glycero-3-phosphocholine (7:0 PC, #850306), 1,2-dioctanoyl-sn-glycero-3-phosphoethanolamine (8:0 PE, #850699), 1,2-dioctanoyl-sn-glycero-3-phosphate sodium salt (8:0 PA, #830842), 1,2-dioctanoyl-sn-glycero-3-phospho-L-serine sodium salt (8:0 PS, #840031), 1,2-dioctanoyl-sn-glycero-3-phospho-(1′-myo-inositol) ammonium salt (8:0 PI, #850181), and cholesterol sulfate (#700016). For the preparation of ALDs, the following long-chain lipids were used: 1,2-dioleoyl-sn-glycero-3-phosphate (18:1 DOPA, #840875), 1,2-dioleoyl-sn-glycero-3-phosphocholine (18:1 DOPC, #850375), 1,2-dioleoyl-sn-glycero-3-phosphoethanolamine (18:1 DOPE, #850725), 1,2-dioleoyl-sn-glycero-3-phospho-L-serine sodium salt (18:1 DOPS, #840035), L-α-phosphatidylinositol sodium salt (soy PI, #840044), glyceryl trioleate (triolein, Merck, #T7140), cholesteryl oleate (Merck, #C9253).

### Yeast strains and culture conditions

The *S. cerevisiae* strains used in this study were WT (BY4742, *MATα leu2Δ0 his3Δ1 lys2Δ0 ura3Δ0*; Euroscarf) and *psd1Δ psd2Δ* (*MATα leu2Δ0 his3Δ200 ura3-52 psd1::KanMX psd2::NatMX*) (39), both carrying a genomically integrated *ERG6-mCherry::HIS3* marker for LD visualization. Strains were confirmed by PCR for deletions and verified for auxotrophic markers. Yeast cells were cultured at 30 °C with shaking in YPD medium (1% yeast extract, 2% peptone, 2% glucose) or in selective synthetic medium (0.67% yeast nitrogen base without amino acids, 2% glucose, and appropriate amino acid dropout supplements). For PE limitation, ethanolamine was supplemented at 2 mM.

### Plasmid construction

The full-length PLIN3 coding sequence and the C-terminal domains of the PLIN proteins were amplified using KAPA HiFi DNA Polymerase (KAPA Biosystems, Roche). C-terminal domains of individual PLINs were as follows: PLIN1 217 to 522, PLIN2 169 to 437, PLIN3 182 to 434, PLIN4 1152 to 1371, PLIN5 166 to 386, lacking the C-terminal mitochondria binding domain. For recombinant protein expression in *Escherichia coli*, PCR products were cloned into the pET16b vector (Novagen, Merck) at the *BamHI* and *NdeI* restriction sites using the Gibson Assembly Cloning Kit (New England Biolabs). Mutant PLIN3 constructs were synthesized and cloned into pET16b by GenScript.

For recombinant protein expression in *Lactococcus lactis*, PCR products were cloned into pNZ16b, derived from the pNZ8048 vector, by insertion of a multiple cloning site including an N-terminal 10xHis tag. DNA sequences encoding the C-terminal domains of PLIN1 and PLIN5 were cloned into pNZ16b between *BamHI* and *HindIII* sites (73).

GFP-tagged PLIN3 was expressed under the control of the ADH1 promoter from the plasmid pGREG576 (pGREG576-ADH1-GFP-PLIN3), as described previously (74). The S346A S347A and S346V S347L mutations were introduced into pGREG576-ADH1-GFP-PLIN3 by site-directed mutagenesis. All constructs were verified by DNA sequencing (Microsynth AG).

### Protein expression and purification

Full-length PLIN3, its C-terminal 4-helix bundle domain (containing residues 182–434), their corresponding S346 S347 mutant variants, and the C-terminal 4-helix bundle domain of PLIN2 (169–437) and PLIN4 (1152-1371) were expressed in *E. coli* BL21 (DE3) cells. Transformed bacteria were cultured in LB medium containing ampicillin at 37 °C to an optical density at 600 nm of 0.5. Protein expression was induced with 0.75 mM IPTG, and cultures were incubated at 30 °C for 2 h before harvesting by centrifugation.

The C-terminal domain of PLIN1(216–522) and PLIN5 (166-386) were expressed in *L. lactis* DML1 cells grown under anaerobic conditions at 28 °C in M17 broth (Formedium) supplemented with 1% glucose and 10 μg/ml chloramphenicol. Protein expression, driven by the pNisA promoter, was induced with 2.5 μg/l nisin at mid-log phase (OD_600_ of 0.5), and cells were further incubated for 2 h at 28 °C (73).

Harvested *E. coli* or *L. lactis* cells were resuspended in lysis buffer containing 50 mM Tris-HCl (pH 7.5), 300 mM NaCl, 20 mM imidazole, 10% glycerol, 1 mM phenylmethylsulfonyl fluoride, and complete EDTA-free protease inhibitor cocktail (PIC; Roche). Cells were disrupted using a Microfluidizer LM10 (Microfluidics) at 800 to 1000 bar pressure, and the clarified lysate was incubated with Ni^2+^-nitrilotriacetic acid (NTA) agarose beads (Qiagen, Hidden) for 2 h, at 4 °C. Bound proteins were eluted with 300 mM imidazole in elution buffer (50 mM Tris-HCl, pH 7.5, 300 mM NaCl, 10% glycerol, 1 mM phenylmethylsulfonyl fluoride, and PIC). Imidazole was removed by buffer exchange into the same buffer lacking imidazole using Zeba spin desalting columns (Thermo Fisher Scientific). Protein concentration was determined by the Lowry method using Folin reagent and bovine serum albumin (BSA) as standard.

### Preparation of artificial lipid droplets

To prepare ALDs, total phospholipids dissolved in chloroform were first transferred to a glass tube and dried completely under a slow stream of nitrogen. The lipid film was then resuspended in 100 μl of buffer B (20 mM Hepes, pH 7.4, 100 mM KCl, 2 mM MgCl_2_). For standard ALDs, 2 mg of phospholipids were used, and neutral lipids, triolein and cholesteryl oleate, were added at a molar ratio of 1:2:1 (phospholipids:triolein:cholesteryl oleate). For preparation of larger ALDs, 0.4 mg of phospholipids were used and mixed with triolein and cholesteryl oleate at a molar ratio of 0.2:2:1. Before addition, cholesteryl oleate was briefly incubated at 50 °C to ensure it was fully liquid. The lipid–aqueous mixture was emulsified by alternating vortexing and resting steps. Standard ALDs were subjected to 24 cycles, followed by a 2 min sonication step, whereas large ALDs were emulsified for 10 cycles without further sonication. The resulting crude emulsions were centrifuged at 1000*g* for 5 min at 4 °C, after which the floating white lipid layer was carefully removed. The remaining fraction was collected and clarified further by centrifugation at 20,000*g* for 5 min at 4 °C to eliminate trace membrane fragments. Dynamic light scattering (NanoLab 3D, LS Instruments AG) analysis confirmed a homogenous size distribution, with standard ALDs displaying a mean diameter of 140 to 180 nm and large ALDs a mean diameter of 320 to 380 nm (75, 76).

#### *In vitro* binding assays

The interaction between purified proteins and either ALDs or short-chain lipids was quantified using microscale thermophoresis (MST) on a Monolith NT.115 system (NanoTemper Technologies). Proteins were labeled at their N-terminal His tags with RED-tris-NTA dye, following the protocol provided by the manufacturer. Serial dilutions of ALDs or short-chain lipids were prepared and combined with a constant concentration of the labeled protein. Samples were loaded into standard MST capillaries, and binding was monitored through changes in fluorescence during thermophoretic movement (Fig. 2*B*). The *K*_*D*_ was determined by plotting the proportion of bound protein against the concentration of the lipid species. Each assay was performed in triplicate, and data fitting was carried out using the *K*_*D*_ model in the MO. Affinity Analysis software package (NanoTemper Technologies).

### Protein–lipid overlay assay

Protein–lipid interactions were assessed using a protein–lipid overlay assay. Lipids prepared as 10 mM stock solutions in chloroform were spotted onto nitrocellulose membranes and air-dried overnight at room temperature. Membranes were blocked with Tris-buffered saline (TBS) containing 3% (w/v) fatty acid–free BSA; Merck, #A8806 and incubated overnight at 4 °C with purified His-tagged proteins (2.5 μg/ml) diluted in TBS supplemented with 0.05% (v/v) Tween-20 and 3% (w/v) fatty acid–free BSA. Membranes were washed with TBS containing 0.05% (v/v) Tween-20 and bound protein was detected by immunoblotting using anti-His antibody (1:1000; Sigma, #SAB1305538) followed by HRP-conjugated anti-mouse secondary antibody (1:10,000; Bio-Rad, #1706516). Signals were developed using SuperSignal West Pico PLUS (Thermo Fisher Scientific, #34580) and imaged with a Fusion FX system (Vilber).

### Flotation assay

PLIN3 or its C-terminal 4-helix bundle domain were incubated with ALDs for 1 h at room temperature. The mixtures were combined with an equal volume of 60% (w/v) sucrose in tris buffer (25 mM Tris, pH 7.5, 50 mM NaCl) to yield a final sucrose concentration of 30%. This was overlaid sequentially with two volumes of 20% sucrose, two volumes of 10% sucrose, and one volume of Tris buffer. Gradients were centrifuged at 177,000 g for 1 h at 20 °C, and four fractions were collected from the top. Protein distribution across the gradient was analyzed by Western blotting using anti-His primary antibody (1:1000; Sigma, #SAB1305538) and HRP-conjugated anti-mouse secondary antibody (1:10,000; Bio-Rad, #1706516). Signals were detected with SuperSignal West Pico Plus (Thermo Fisher Scientific, #34580) and imaged using a Fusion FX Imager (Vilber; vilber.com). Band intensities were quantified using ImageJ software (Fiji v2.14.0; imagej.net).

### CD Spectroscopy

Far-UV CD measurements were carried out on a Chirascan-Q100 spectropolarimeter (Applied Photophysics Ltd) using 10 mm path length quartz cuvettes. Protein samples were diluted in 10 mM potassium phosphate buffer (pH 7.5) to a final concentration of 0.01 mg/ml. Spectra were recorded over the 190 to 260 nm range with 1 nm increments and a 1 nm bandwidth. For each sample, five consecutive scans were acquired, averaged, and expressed in millidegrees, then converted to mean residue ellipticity. Baseline correction was applied for buffer subtraction. Processed spectra were visualized using GraphPad Prism (GraphPad Software; graphpad.com).

### Monitoring LD localization in *S. cerevisiae*

*S. cerevisiae* WT and *psd1Δ psd2Δ* double mutant strains co-expressing GFP-tagged PLIN3 or the S346V S347L variant along with the LD marker Erg6-mCherry were cultured at 30 °C in medium supplemented with 2 mM ethanolamine. Cells were grown to OD_600_ of 1, harvested by centrifugation, washed twice with ethanolamine-free synthetic medium, and resuspended in the same medium lacking ethanolamine to induce PE depletion. Samples were collected immediately after transfer (0 h), at 2 h intervals up to 8 h, and again at 24 h for fluorescence microscopy analysis. To evaluate whether restoration of PE levels rescues PLIN3 LD localization, *psd1Δ psd2Δ* cells previously incubated in ethanolamine-free medium for 24 h were collected, washed once with ethanolamine-supplemented medium, and resuspended in fresh medium containing 2 mM ethanolamine. Cells were incubated at 30 °C, and samples were collected at defined time point’s post-resupplementation for fluorescence microscopy analysis.

### Fluorescence microscopy

Fluorescence images were acquired using a Visitron spinning disk CSU-W1 system (Visitron Systems) equipped with a Nikon Ti-E inverted microscope, a CSU-W1 spinning disk head (Yokogawa), a Hamamatsu Orca Quest (C15550-20UP) qCMOS camera, and a Plan Apo 100× NA 1.3 oil objective (Nikon).

Image processing and quantification were performed with ImageJ software (Fiji v2.14.0). Individual cells were manually selected as regions of interest (ROIs), and total GFP fluorescence within each cellular ROI was measured after background subtraction. Punctate GFP signals colocalizing with Erg6-mCherry–labeled LDs were identified using Gaussian blur filtering followed by difference-of-Gaussians processing to generate punctate ROIs. The integrated fluorescence intensity of punctate ROIs was expressed as a percentage of total cellular GFP fluorescence to quantify PLIN3 localization to LDs.

The number and size of LDs per cell were quantified from fluorescence microscopy images using Biodock AI (Biodock AI Software Platform, 2024, www.biodock.ai), an artificial intelligence–based image analysis platform. Prior to analysis, the AI model was trained on representative image sets to accurately recognize and segment LDs marked by Erg6-mCherry. Following training, images were processed through the platform for automated detection and segmentation of individual LDs. For each cell, the total number of LDs and the area of each droplet were measured. Quantitative data were exported for subsequent statistical analysis. n > 60 cells/strain from five independent experiments.

### Protein stability assay by cycloheximide chase

Yeast strains expressing GFP-tagged PLIN3 or the S346V S347L variant were grown at 30 °C in synthetic complete medium containing 2% glucose and 2 mM ethanolamine to mid-log phase. Cells were then harvested, washed, and transferred to ethanolamine-free medium to induce PE depletion. At the time of transfer, cycloheximide (50 μg/ml) was added to inhibit protein synthesis in treated samples, while control samples received no cycloheximide. Aliquots were collected immediately after transfer (0 h), after 1h, and then every 2 h up to 8 h, and again after 24 h. Protein levels were analyzed by western blotting using anti-PLIN3 antibodies (1:2000; Proteintech, Rosemont, Il, USA, #10694-1-AP) to detect PLIN3 (74 kDa with GFP tag) and its mutant variant. Phosphoglycerate kinase 1 (Pgk1, 44 kDa) was used as a stable loading control (anti-Pgk1; 1:2000, Invitrogen #459250), and alpha-1,3-mannosyltransferase (Mnn1, 88 kDa) was monitored with anti-Mnn1 antibodies (1:2000; MyBioSource #MBS7155562) as a short–half-life control to verify cycloheximide chase efficiency. Changes in protein abundance over time were assessed to determine protein stability during PE depletion. Band intensities were quantified using ImageJ software (Fiji v2.14.0).

### Statistical analyses

Data were statistically analyzed using GraphPad Prism 10.4.2 software. Data normality was assessed using the D’Agostino–Pearson omnibus test. Parametric tests were applied to datasets with a normal (Gaussian) distribution, whereas nonparametric tests were used when the normality criterion was not met. The specific statistical tests applied in each case are indicated in the figure legends. Statistical significance is denoted in the graphs by asterisks and in Table S1.

### Molecular dynamics simulations

Two membrane systems were constructed using CHARMM-GUI Membrane Builder (77, 78). Each system contained 252 lipids (126 lipids per leaflet). The PC-only system consisted of 90% phosphatidylcholine (PC) composed of DOPC and POPC in a 1:1 ratio (60 molecules each per leaflet) and 10% triacylglycerol (POLT, six molecules per leaflet). The PE-containing system consisted of 70% PC (DOPC:POPC 1:1; 48 molecules each per leaflet), 20% phosphatidylethanolamine (PE) (DOPE:POPE 1:1; 12 molecules each per leaflet), and 10% TAG (POLT, six molecules per leaflet). The conserved cleft region of PLIN3 (residues 233–265, 337–349, and 412–429) was extracted from the AlphaFold3 prediction of the full C-terminal domain (residues 183–434) (79). The three segments were connected with short flexible GGG linkers using Modeller (80). The protein construct was initially positioned 20 Å above the membrane surface. The systems were solvated using a minimum of 22.5 Å water layers on each side of the membrane with the TIP3P water model, and neutralized with sodium and chloride ions (0.15 M NaCl).

Simulations were performed with OpenMM 8.1 using the CHARMM36m force field (81, 82). Systems were energy-minimized for 5000 steps and equilibrated using the standard six-stage CHARMM-GUI protocol with gradually increasing timestep (1–4 fs). Following this procedure, an additional 1 ns equilibration was performed in the NPT ensemble at 298 K and 1 bar using a 2 fs timestep, while applying positional restraints (500 kJ mol^-1^ nm^-2^) to the protein atoms. Production simulations were subsequently run for 250 ns in the NPT ensemble at 298 K and 1 bar, using a Langevin integrator with a 4 fs timestep enabled by hydrogen mass repartitioning. Pressure was maintained using a Monte Carlo barostat, and long-range electrostatics were treated with the particle mesh Ewald method (83). The final 150 ns (100–250 ns) of each simulation were used for analysis.

Trajectory analyses were performed using MDAnalysis package (84, 85). For each frame, the minimum distance between any protein atom and any phospholipid phosphorus atom was calculated. A protein–membrane contact was defined as a minimum distance of ≤ 7 Å. Contact frequency was calculated as the percentage of frames in which at least one such contact occurred. Probability density distributions were computed from the minimum-distance values of frames containing contacts. For visualization, raw distance time series were overlaid with Savitzky–Golay smoothed traces (window length = 51 frames, polynomial order = 2). Structural figures and molecular visualizations were prepared using UCSF ChimeraX (86).

### *In silico* ligand docking

Docking analyses were performed with AutoDock Vina (43, 87) using an AlphaFold3-predicted structure of PLIN3, as no experimental structure is currently available (79). Lipid models were retrieved from PubChem or ChemSpider (88), converted to PDB format in PyMOL (v2.5.4, Schrödinger, LLC.), and prepared together with the protein in AutoDock Tools. The protein structure was set as the macromolecule target and lipids as ligands, with all files saved in PDBQT format (89).

The docking grid was positioned over the C-terminal 4-helix bundle and adjusted to cover the entire domain. Simulations were run with standard Vina settings, and docking results were examined in UCSF ChimeraX to visualize and compare predicted binding poses (86).

The evolutionary conservation pattern of the C-terminal 4-helix bundle domain of PLIN3 was evaluated using the ConSurf server (90). The AlphaFold3-predicted structure of full-length PLIN3 served as the structural template, from which the C-terminal domain was analyzed. ConSurf identified homologous sequences, performed multiple sequence alignment, and calculated residue conservation scores of representative orthologs. The resulting scores were mapped onto the domain structure to highlight conserved surface features.

## Data availability

All data is contained within the manuscript.

## Supporting information

This article contains supporting information. Supplementary Figures S1-S3, Table S1.

## Declaration of generative AI and AI-assisted technologies in the writing process

During the preparation of this work the authors used AI-assisted technologies to improve the readability and language of the manuscript. After using this tool, the authors reviewed and edited the content as needed and take full responsibility for the content of the published article.

## Conflict of interest

The authors declare that they have no conflicts of interest with the contents of this article.
