# Supplementary material for: Phosphatidylethanolamine binds to human perilipins via a hydrophobic cleft in their 4-helix bundle domain for lipid droplet targeting
Source: J Biol Chem. 2026 Jun 19;302(8):113268. doi: 10.1016/j.jbc.2026.113268 (PMC13382019; doi:10.1016/j.jbc.2026.113268)
Supplement: Supplementary Data [file mmc1.pdf]

Supplementary Data

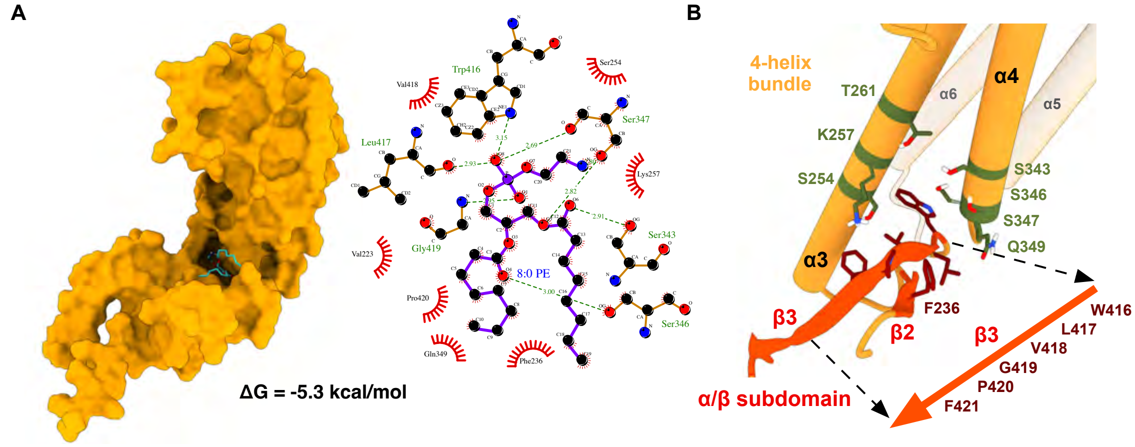

C

AAs mutation to:	ΔG (kcal/mol) ALA	LEU
W416	- 6.3	- 5.6
S347	- 5.0	- 4.1
S347 W416	- 5.9	- 4.4
S346 S347	- 5.4	- 4.0
F236 S346 S347	- 4.9	- 3.8
F236 S346 S347 W416	- 5.8	- 4.0

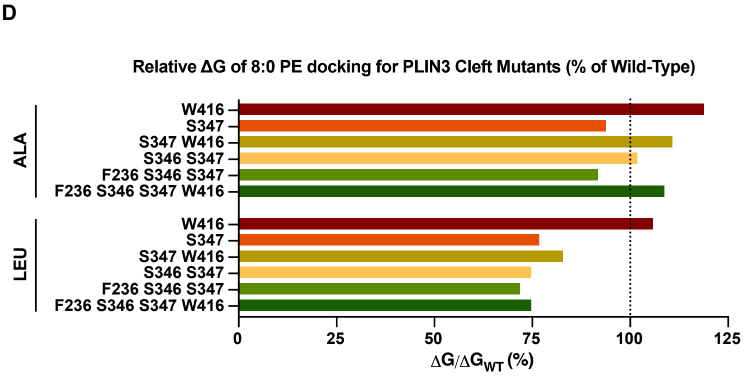

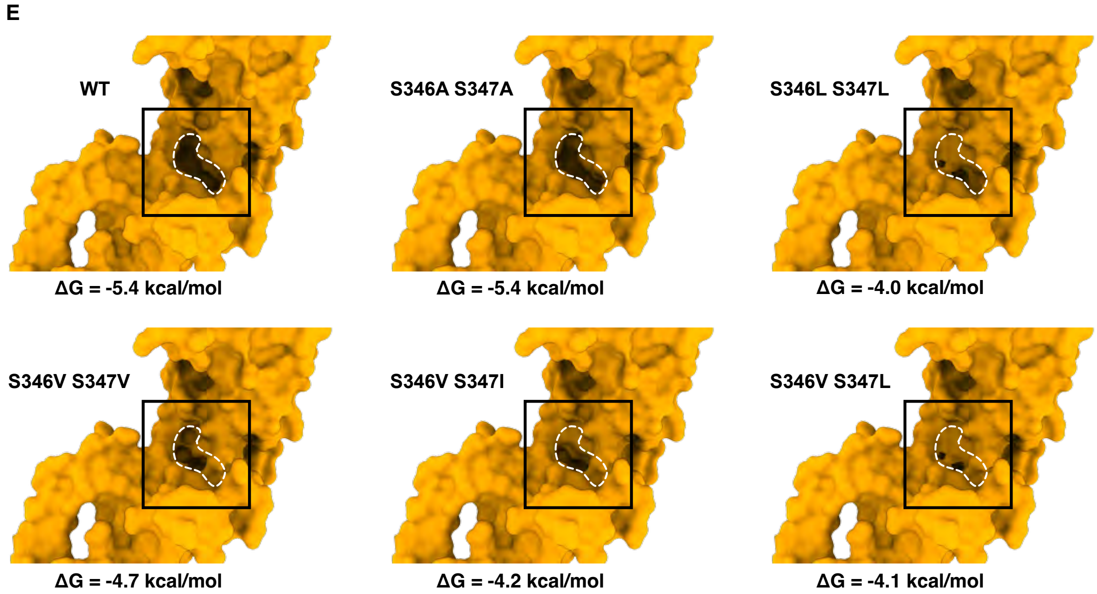

Figure S1. *In silico* modeling and docking analysis of mutations in the central hydrophobic cleft of the 4-helix domain of PLIN3.

A) Structural representation of the wild-type PLIN3 4-helix bundle domain with docked 8:0 phosphatidylethanolamine (8:0 PE). The predicted binding free energy (ΔG) derived from docking simulations is indicated. A corresponding 2D interaction map generated by LigPlot

illustrates key contacts between the ligand and surrounding residues within the central cleft. Complementary analysis using the protein–ligand interaction profiler (PLIP) confirmed non-covalent interactions, including hydrogen bonds and hydrophobic contacts.

B) Cartoon representation of the PLIN3 C-terminal domain highlighting its two-part organization, consisting of a four-helix bundle (orange) and an α/β subdomain (red). Helices are represented as cylinders and β-strands as arrows. Polar residues (green) line the rim of the hydrophobic cleft on the 4-helix bundle side, whereas conserved hydrophobic residues (brown) within the β2 and β3 strands of the α/β subdomain form the opposing surface of the cleft. For clarity, the β3 strand is additionally depicted aside the figure to better illustrate the position of the corresponding conserved hydrophobic residues. This representation highlights the amphipathic architecture of the cleft and its potential role in ligand accommodation.

C) Predicted binding energies (ΔG values) of a panel of *in silico*-generated mutations in the hydrophobic cleft of PLIN3 to short-chain 8:0 PE. Each indicated residue was substituted by either alanine (Ala) or leucine (Leu), and docking simulations were performed for each variant.

D) Relative binding affinities of mutations in the hydrophobic cleft of the 4-helix domain from panel B expressed as a percentage of wild-type binding. The values represent ΔG of each mutant normalized to the ΔG of wild-type (set as 100%, indicated by the dotted line). While Ala substitutions had minimal impact on predicted binding energies, serine-to-leucine mutations consistently reduced binding affinity by about 25%. This suggests that an increased size of the sidechain at these positions may impair access of the ligand to the hydrophobic cleft.

E) Analysis of double substitutions of S346 and S347 with the indicated amino acids, highlighting the predicted structural changes in the hydrophobic cleft. While substitutions with Ala preserved an open cleft structure as in the wild-type, substitutions by bulkier side chains such as Leu resulted in cleft closure, potentially occluding access of the ligand to the binding pocket. The ΔG values below each model indicate the docking energies of 8:0 PE for the corresponding mutant variants. All docking performed with AutoDock Vina; structures from AlphaFold3.

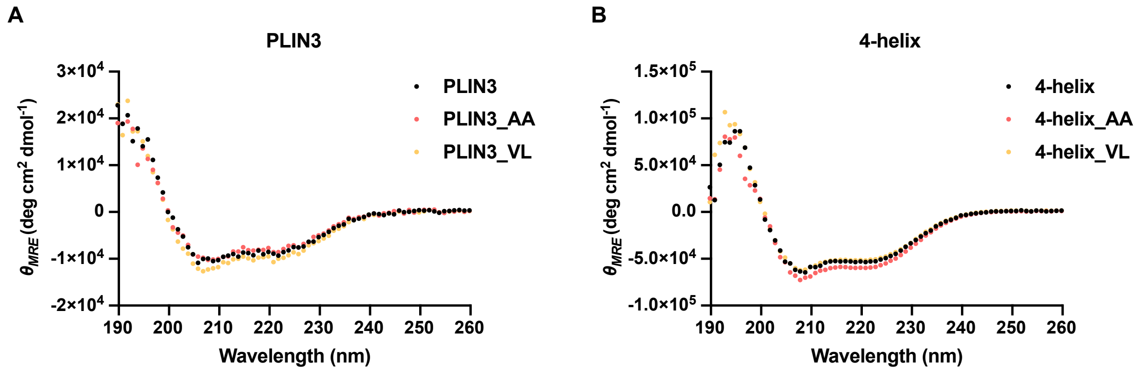

Figure S2. Circular dichroism of wild-type and mutant 4-helix bundle domains of PLIN3. Far-UV circular dichroism (CD) spectra of wild-type and mutant versions of the full-length (panel A) and the 4-helix bundle domain of PLIN3 (panel B) are provided to confirm their structural integrity. Spectra were recorded as described in Materials and Methods, showing characteristic α-helical signatures (negative peaks at 208 and 222 nm) with minimal deviations in mutants, indicating that the mutations affect lipid binding through the hydrophobic cleft without affecting the overall fold of the 4-helix bundle domain of PLIN3. Mean residue ellipticity is plotted *vs* wavelength, n=3 scans/sample.

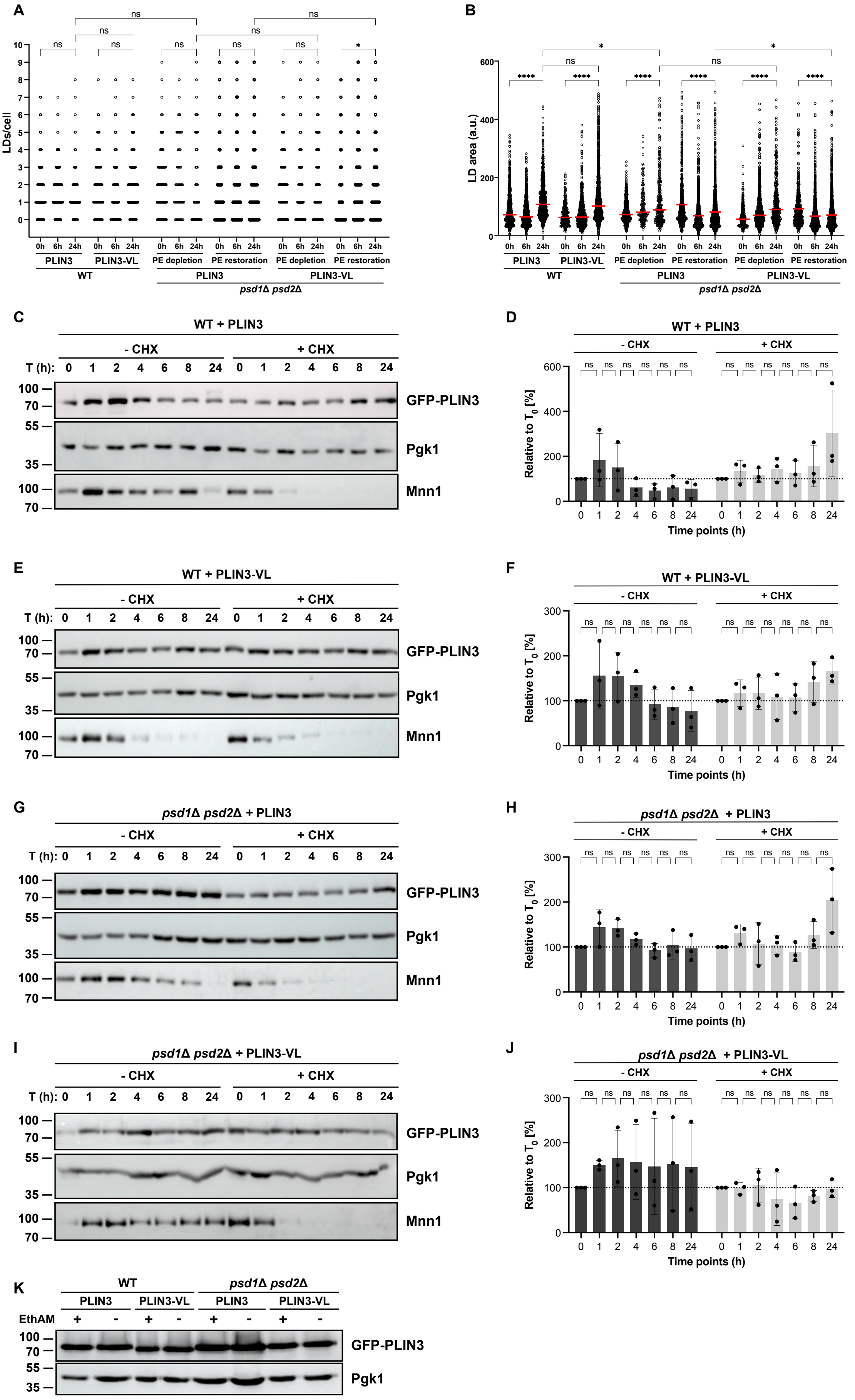
A
LDs/cell
0h 6h 24h
PLIN3
PLIN3-VL
WT
PE depletion
PE restoration
PLIN3
PLIN3-VL
psd1Δ psd2Δ
ns
*
B
LD area (a.u.)
****
C
WT + PLIN3
- CHX
+ CHX
T (h): 0 1 2 4 6 8 24 0 1 2 4 6 8 24
GFP-PLIN3
Pgk1
Mnn1
D
Relative to T0 [%]
Time points (h)
E
WT + PLIN3-VL
F
G
psd1Δ psd2Δ + PLIN3
H
I
psd1Δ psd2Δ + PLIN3-VL
J
K
EthAM
+ -
100
70
55
35

Figure S3. PLIN3 and the S346V S347L mutant variant are stable when expressed in *S. cerevisiae* cells.

A, B) Quantification of LD number per cell (panel A) and individual LD area (panel B) in wild-type (WT) and *psd1Δ psd2Δ* double mutant cells expressing GFP-tagged PLIN3 or the PLIN3-S346V S347L (PLIN3-VL) mutant version. Cells were grown in ethanolamine-supplemented medium, then shifted to ethanolamine-free medium (Time 0 h), and followed for 24 h to monitor protein localization under PE-limiting conditions. *psd1Δ psd2Δ* cells were analyzed under PE depletion (−EthAM) and subsequently transferred back to ethanolamine-supplemented medium (+EthAM) to restore PE levels. Data from five independent experiments (panel A, n > 60 cells; panel B, n > 200 LDs, red line indicates median). *p < 0.05; ****p < 0.0001; ns, not significant (Kruskal–Wallis test followed by Dunn's multiple comparison post hoc test).

C-J) The stability of the GFP-tagged PLIN3 (74 kDa) (panels C, G) or the PLIN3-S346V S347L (PLIN3-VL) (panels E, I) mutant version expressed in wild-type cells (WT; panels C, E) or *psd1Δ psd2Δ* double mutant cells (panel G, I) was assessed by cycloheximide chase. Cells expressing the indicated protein were diluted in fresh medium with or without cycloheximide (CHX, 50 µg/ml) and samples were removed at the indicated time points. The relative abundance of the indicated protein was assessed by western blotting. Phosphoglycerate kinase 1 (Pgk1, 44 kDa) was used as a loading control, and alpha-1,3-mannosyltransferase (Mnn1, 88 kDa) as a short half-life control protein to verify cycloheximide chase efficiency. Band intensities were quantified using ImageJ/Fiji and plotted as relative abundance normalized to Pgk1 and time 0 (mean ± SD from three independent experiments; no significant degradation observed, p > 0.05 by two-way ANOVA with Tukey's post hoc test).

K) Western blot analysis of GFP-tagged PLIN3 and PLIN3-S346V S347L (PLIN3-VL) expressed in WT or *psd1Δ psd2Δ* double mutant cells grown in ethanolamine-supplemented medium (+EthAM) or after transfer to ethanolamine-free medium (−EthAM). Pgk1 was used as a loading control.

Table S1. Complete report of p values from the statistical analyses.

Figure 2A. Phospholipid titration - PE	
Tukey's multiple comparisons test	Adjusted P Value
PLIN3:0mol% vs. PLIN3:2.5mol%	>0.9999
PLIN3:0mol% vs. PLIN3:5mol%	>0.9999
PLIN3:0mol% vs. PLIN3:10mol%	0.9896
PLIN3:0mol% vs. PLIN3:20mol%	0.9994
PLIN3:0mol% vs. 4helix:0mol%	>0.9999
PLIN3:0mol% vs. 4helix:2.5mol%	<0.0001
PLIN3:0mol% vs. 4helix:5mol%	<0.0001
PLIN3:0mol% vs. 4helix:10mol%	<0.0001
PLIN3:0mol% vs. 4helix:20mol%	<0.0001
PLIN3:2.5mol% vs. PLIN3:5mol%	>0.9999
PLIN3:2.5mol% vs. PLIN3:10mol%	0.9798
PLIN3:2.5mol% vs. PLIN3:20mol%	0.9981
PLIN3:2.5mol% vs. 4helix:0mol%	>0.9999
PLIN3:2.5mol% vs. 4helix:2.5mol%	<0.0001
PLIN3:2.5mol% vs. 4helix:5mol%	<0.0001
PLIN3:2.5mol% vs. 4helix:10mol%	<0.0001
PLIN3:2.5mol% vs. 4helix:20mol%	<0.0001
PLIN3:5mol% vs. PLIN3:10mol%	0.9896
PLIN3:5mol% vs. PLIN3:20mol%	0.9994
PLIN3:5mol% vs. 4helix:0mol%	>0.9999
PLIN3:5mol% vs. 4helix:2.5mol%	<0.0001
PLIN3:5mol% vs. 4helix:5mol%	<0.0001
PLIN3:5mol% vs. 4helix:10mol%	<0.0001
PLIN3:5mol% vs. 4helix:20mol%	<0.0001
PLIN3:10mol% vs. PLIN3:20mol%	>0.9999
PLIN3:10mol% vs. 4helix:0mol%	0.8930
PLIN3:10mol% vs. 4helix:2.5mol%	<0.0001
PLIN3:10mol% vs. 4helix:5mol%	<0.0001
PLIN3:10mol% vs. 4helix:10mol%	<0.0001
PLIN3:10mol% vs. 4helix:20mol%	<0.0001
PLIN3:20mol% vs. 4helix:0mol%	0.9728
PLIN3:20mol% vs. 4helix:2.5mol%	<0.0001
PLIN3:20mol% vs. 4helix:5mol%	<0.0001
PLIN3:20mol% vs. 4helix:10mol%	<0.0001
PLIN3:20mol% vs. 4helix:20mol%	<0.0001
4helix:0mol% vs. 4helix:2.5mol%	<0.0001
4helix:0mol% vs. 4helix:5mol%	<0.0001
4helix:0mol% vs. 4helix:10mol%	<0.0001
4helix:0mol% vs. 4helix:20mol%	<0.0001
4helix:2.5mol% vs. 4helix:5mol%	0.9989
4helix:2.5mol% vs. 4helix:10mol%	0.9798
4helix:2.5mol% vs. 4helix:20mol%	0.2153
4helix:5mol% vs. 4helix:10mol%	>0.9999
4helix:5mol% vs. 4helix:20mol%	0.5891
4helix:10mol% vs. 4helix:20mol%	0.8006

Figure 2A. Phospholipid titration			
Tukey's multiple	Adjusted P Value		
	PA	PS	PI
PLIN3:0mol% vs. PLIN3:2.5mol%	0.9993	0.3999	0.4059
PLIN3:0mol% vs. PLIN3:5mol%	0.9996	0.3435	0.9989
PLIN3:0mol% vs. PLIN3:10mol%	>0.9999	0.9966	0.0014
PLIN3:0mol% vs. 4helix:0mol%	0.2208	0.2804	0.0864
PLIN3:0mol% vs. 4helix:2.5mol%	0.2814	0.3906	0.1721
PLIN3:0mol% vs. 4helix:5mol%	0.2060	>0.9999	0.0870
PLIN3:0mol% vs. 4helix:10mol%	0.3516	0.0005	0.0901
PLIN3:2.5mol% vs. PLIN3:5mol%	0.9651	>0.9999	0.1750
PLIN3:2.5mol% vs. PLIN3:10mol%	>0.9999	0.7835	<0.0001
PLIN3:2.5mol% vs. 4helix:0mol%	0.0903	>0.9999	0.9718
PLIN3:2.5mol% vs. 4helix:2.5mol%	0.1195	>0.9999	0.9987
PLIN3:2.5mol% vs. 4helix:5mol%	0.0834	0.2772	0.9723
PLIN3:2.5mol% vs. 4helix:10mol%	0.1560	<0.0001	0.9752
PLIN3:5mol% vs. PLIN3:10mol%	0.9936	0.7221	0.0041
PLIN3:5mol% vs. 4helix:0mol%	0.4406	>0.9999	0.0300
PLIN3:5mol% vs. 4helix:2.5mol%	0.5307	>0.9999	0.0635
PLIN3:5mol% vs. 4helix:5mol%	0.4169	0.2334	0.0302
PLIN3:5mol% vs. 4helix:10mol%	0.6230	<0.0001	0.0314
PLIN3:10mol% vs. 4helix:0mol%	0.1436	0.6400	<0.0001
PLIN3:10mol% vs. 4helix:2.5mol%	0.1870	0.7741	<0.0001
PLIN3:10mol% vs. 4helix:5mol%	0.1333	0.9773	<0.0001
PLIN3:10mol% vs. 4helix:10mol%	0.2395	0.0001	<0.0001
4helix:0mol% vs. 4helix:2.5mol%	>0.9999	>0.9999	0.9999
4helix:0mol% vs. 4helix:5mol%	>0.9999	0.1863	>0.9999
4helix:0mol% vs. 4helix:10mol%	>0.9999	<0.0001	>0.9999
4helix:2.5mol% vs. 4helix:5mol%	>0.9999	0.2699	0.9999
4helix:2.5mol% vs. 4helix:10mol%	>0.9999	<0.0001	>0.9999
4helix:5mol% vs. 4helix:10mol%	>0.9999	0.0007	>0.9999

Figure 3C. Flotation assay	
Bonferroni's multiple test	Adjusted P Value
PC+PE vs PC	0.0012
ER vs ERwoPE	<0.0001

Figure 3D. ALDs PC vs PC/PE		
Bonferroni's multiple test	Adjusted P Value	
	small ALDs	large ALDs
PLIN3	>0.9999	>0.9999

Figure 3E. ALDs ER vs ER w/o PE		
Bonferroni's multiple test	Adjusted P Value	
	small ALDs	large ALDs
PLIN3	>0.9999	>0.9999
4helix	0.0010	0.0005

Figure 2B. Short chain phospholipid	
Tukey's multiple comparisons test	Adjusted P Value
4-helix:7:0 PC vs. 4-helix:8:0 PE	<0.0001
4-helix:7:0 PC vs. 4-helix:8:0 PS	0.8476
4-helix:7:0 PC vs. 4-helix:8:0 PI	0.9174
4-helix:7:0 PC vs. PLIN3:7:0 PC	0.8405
4-helix:7:0 PC vs. PLIN3:8:0 PE	0.0623
4-helix:7:0 PC vs. PLIN3:8:0 PA	0.7745
4-helix:7:0 PC vs. PLIN3:8:0 PS	0.9708
4-helix:7:0 PC vs. PLIN3:8:0 PI	0.8405
4-helix:8:0 PE vs. 4-helix:8:0 PS	<0.0001
4-helix:8:0 PE vs. 4-helix:8:0 PI	<0.0001
4-helix:8:0 PE vs. PLIN3:7:0 PC	<0.0001
4-helix:8:0 PE vs. PLIN3:8:0 PE	<0.0001
4-helix:8:0 PE vs. PLIN3:8:0 PA	<0.0001
4-helix:8:0 PE vs. PLIN3:8:0 PS	<0.0001
4-helix:8:0 PE vs. PLIN3:8:0 PI	<0.0001
4-helix:8:0 PS vs. 4-helix:8:0 PI	0.9295
4-helix:8:0 PS vs. PLIN3:7:0 PC	0.9927
4-helix:8:0 PS vs. PLIN3:8:0 PE	0.0423
4-helix:8:0 PS vs. PLIN3:8:0 PA	0.6329
4-helix:8:0 PS vs. PLIN3:8:0 PS	0.8191
4-helix:8:0 PS vs. PLIN3:8:0 PI	0.9927
4-helix:8:0 PI vs. PLIN3:7:0 PC	0.9222
4-helix:8:0 PI vs. PLIN3:8:0 PE	0.0507
4-helix:8:0 PI vs. PLIN3:8:0 PA	0.6967
4-helix:8:0 PI vs. PLIN3:8:0 PS	0.8884
4-helix:8:0 PI vs. PLIN3:8:0 PI	0.9222
PLIN3:7:0 PC vs. PLIN3:8:0 PE	<0.0001
PLIN3:7:0 PC vs. PLIN3:8:0 PA	0.2512
PLIN3:7:0 PC vs. PLIN3:8:0 PS	0.8556
PLIN3:7:0 PC vs. PLIN3:8:0 PI	>0.9999
PLIN3:7:0 PC vs. PLIN3:Cholest sulph.	0.1740
PLIN3:8:0 PE vs. PLIN3:8:0 PA	<0.0001
PLIN3:8:0 PE vs. PLIN3:8:0 PS	<0.0001
PLIN3:8:0 PE vs. PLIN3:8:0 PI	<0.0001
PLIN3:8:0 PE vs. PLIN3:Cholest sulph.	<0.0001
PLIN3:8:0 PA vs. PLIN3:8:0 PS	0.8295
PLIN3:8:0 PA vs. PLIN3:8:0 PI	0.2512
PLIN3:8:0 PA vs. PLIN3:Cholest sulph.	0.9998
PLIN3:8:0 PS vs. PLIN3:8:0 PI	0.8556
PLIN3:8:0 PS vs. PLIN3:Cholest sulph.	0.7006
PLIN3:8:0 PI vs. PLIN3:Cholest sulph.	0.1740

Figure 5B. S346 S347 mutants - ALDs

Tukey's multiple comparisons test	Adjusted P Value
4helix-SS_VL:ALD-PC vs. 4helix-SS_VL:ALD-PC/PE	>0.9999
4helix-SS_VL:ALD-PC vs. 4helix-SS_AA:ALD-PC	>0.9999
4helix-SS_VL:ALD-PC vs. 4helix-SS_AA:ALD-PC/PE	0.9966
4helix-SS_VL:ALD-PC vs. 4helix:ALD-PC	>0.9999
4helix-SS_VL:ALD-PC vs. 4helix:ALD-PC/PE	<0.0001
4helix-SS_VL:ALD-PC vs. PLIN3-SS_VL:ALD-PC	0.0043
4helix-SS_VL:ALD-PC vs. PLIN3-SS_VL:ALD-PC/PE	0.1250
4helix-SS_VL:ALD-PC vs. PLIN3-SS_AA:ALD-PC	0.2555
4helix-SS_VL:ALD-PC vs. PLIN3-SS_AA:ALD-PC/PE	0.0069
4helix-SS_VL:ALD-PC vs. PLIN3:ALD-PC	0.9966
4helix-SS_VL:ALD-PC vs. PLIN3:ALD-PC/PE	0.9223
4helix-SS_VL:ALD-PC/PE vs. 4helix-SS_AA:ALD-PC	>0.9999
4helix-SS_VL:ALD-PC/PE vs. 4helix-SS_AA:ALD-PC/PE	0.9966
4helix-SS_VL:ALD-PC/PE vs. 4helix:ALD-PC	>0.9999
4helix-SS_VL:ALD-PC/PE vs. 4helix:ALD-PC/PE	<0.0001
4helix-SS_VL:ALD-PC/PE vs. PLIN3-SS_VL:ALD-PC	0.0043
4helix-SS_VL:ALD-PC/PE vs. PLIN3-SS_VL:ALD-PC/PE	0.1250
4helix-SS_VL:ALD-PC/PE vs. PLIN3-SS_AA:ALD-PC	0.2555
4helix-SS_VL:ALD-PC/PE vs. PLIN3-SS_AA:ALD-PC/PE	0.0069
4helix-SS_VL:ALD-PC/PE vs. PLIN3:ALD-PC	0.9966
4helix-SS_VL:ALD-PC/PE vs. PLIN3:ALD-PC/PE	0.9223
4helix-SS_AA:ALD-PC vs. 4helix-SS_AA:ALD-PC/PE	>0.9999
4helix-SS_AA:ALD-PC vs. 4helix:ALD-PC	>0.9999
4helix-SS_AA:ALD-PC vs. 4helix:ALD-PC/PE	<0.0001
4helix-SS_AA:ALD-PC vs. PLIN3-SS_VL:ALD-PC	0.0110
4helix-SS_AA:ALD-PC vs. PLIN3-SS_VL:ALD-PC/PE	0.2555
4helix-SS_AA:ALD-PC vs. PLIN3-SS_AA:ALD-PC	0.4600
4helix-SS_AA:ALD-PC vs. PLIN3-SS_AA:ALD-PC/PE	0.0173
4helix-SS_AA:ALD-PC vs. PLIN3:ALD-PC	>0.9999
4helix-SS_AA:ALD-PC vs. PLIN3:ALD-PC/PE	0.9905
4helix-SS_AA:ALD-PC/PE vs. 4helix:ALD-PC	0.9966
4helix-SS_AA:ALD-PC/PE vs. 4helix:ALD-PC/PE	<0.0001
4helix-SS_AA:ALD-PC/PE vs. PLIN3-SS_VL:ALD-PC	0.0420
4helix-SS_AA:ALD-PC/PE vs. PLIN3-SS_VL:ALD-PC/PE	0.5821
4helix-SS_AA:ALD-PC/PE vs. PLIN3-SS_AA:ALD-PC	0.8147
4helix-SS_AA:ALD-PC/PE vs. PLIN3-SS_AA:ALD-PC/PE	0.0642
4helix-SS_AA:ALD-PC/PE vs. PLIN3:ALD-PC	>0.9999
4helix-SS_AA:ALD-PC/PE vs. PLIN3:ALD-PC/PE	>0.9999
4helix:ALD-PC vs. 4helix:ALD-PC/PE	<0.0001
4helix:ALD-PC vs. PLIN3-SS_VL:ALD-PC	0.0043
4helix:ALD-PC vs. PLIN3-SS_VL:ALD-PC/PE	0.1250
4helix:ALD-PC vs. PLIN3-SS_AA:ALD-PC	0.2555
4helix:ALD-PC vs. PLIN3-SS_AA:ALD-PC/PE	0.0069
4helix:ALD-PC vs. PLIN3:ALD-PC	0.9966
4helix:ALD-PC vs. PLIN3:ALD-PC/PE	0.9223
4helix:ALD-PC/PE vs. PLIN3-SS_VL:ALD-PC	<0.0001
4helix:ALD-PC/PE vs. PLIN3-SS_VL:ALD-PC/PE	<0.0001
4helix:ALD-PC/PE vs. PLIN3-SS_AA:ALD-PC	<0.0001
4helix:ALD-PC/PE vs. PLIN3-SS_AA:ALD-PC/PE	<0.0001
4helix:ALD-PC/PE vs. PLIN3:ALD-PC	<0.0001
4helix:ALD-PC/PE vs. PLIN3:ALD-PC/PE	<0.0001
PLIN3-SS_VL:ALD-PC vs. PLIN3-SS_VL:ALD-PC/PE	0.9223
PLIN3-SS_VL:ALD-PC vs. PLIN3-SS_AA:ALD-PC	0.7434
PLIN3-SS_VL:ALD-PC vs. PLIN3-SS_AA:ALD-PC/PE	>0.9999
PLIN3-SS_VL:ALD-PC vs. PLIN3:ALD-PC	0.0420
PLIN3-SS_VL:ALD-PC vs. PLIN3:ALD-PC/PE	0.1250
PLIN3-SS_VL:ALD-PC/PE vs. PLIN3-SS_AA:ALD-PC	>0.9999

Figure 5B. S346 S347 mutants - ALDs (continued)

Tukey's multiple comparisons test	Adjusted P Value
PLIN3-SS_VL:ALD-PC/PE vs. PLIN3-SS_AA:ALD-PC/PE	0.9685
PLIN3-SS_VL:ALD-PC/PE vs. PLIN3:ALD-PC	0.5821
PLIN3-SS_VL:ALD-PC/PE vs. PLIN3:ALD-PC/PE	0.8750
PLIN3-SS_AA:ALD-PC vs. PLIN3-SS_AA:ALD-PC/PE	0.8464
PLIN3-SS_AA:ALD-PC vs. PLIN3:ALD-PC	0.8147
PLIN3-SS_AA:ALD-PC vs. PLIN3:ALD-PC/PE	0.9781
PLIN3-SS_AA:ALD-PC/PE vs. PLIN3:ALD-PC	0.0642
PLIN3-SS_AA:ALD-PC/PE vs. PLIN3:ALD-PC/PE	0.1812
PLIN3:ALD-PC vs. PLIN3:ALD-PC/PE	>0.9999

Figure 5C. S346 S347 mutants - short chain PE

Tukey's multiple comparisons test	Adjusted P Value
4helix-SS_VL vs. 4helix-SS_AA	0.8484
4helix-SS_VL vs. 4helix	<0.0001
4helix-SS_VL vs. PLIN3-SS_VL	0.9998
4helix-SS_VL vs. PLIN3-SS_AA	0.9647
4helix-SS_VL vs. PLIN3	0.8423
4helix-SS_AA vs. 4helix	<0.0001
4helix-SS_AA vs. PLIN3-SS_VL	0.7240
4helix-SS_AA vs. PLIN3-SS_AA	0.9988
4helix-SS_AA vs. PLIN3	>0.9999
4helix vs. PLIN3-SS_VL	<0.0001
4helix vs. PLIN3-SS_AA	<0.0001
4helix vs. PLIN3	<0.0001
PLIN3-SS_VL vs. PLIN3-SS_AA	0.8972
PLIN3-SS_VL vs. PLIN3	0.7163
PLIN3-SS_AA vs. PLIN3	0.9986

Figure 6E. PLIN3 in WT

Tukey's multiple comparisons test		Adjusted P Value
A-B	0h vs. 6h	0.0266
A-C	0h vs. 24h	<0.0001
A-D	0h vs. 0h	0.9830
A-E	0h vs. 6h	0.9755
A-F	0h vs. 24h	0.7399
B-C	6h vs. 24h	0.0026
B-D	6h vs. 0h	0.1116
B-E	6h vs. 6h	0.0047
B-F	6h vs. 24h	0.0010
C-D	24h vs. 0h	<0.0001
C-E	24h vs. 6h	<0.0001
C-F	24h vs. 24h	<0.0001
D-E	0h vs. 6h	0.7209
D-F	0h vs. 24h	0.3442
E-F	6h vs. 24h	0.9864

Letters indicate data groups as arranged on the x-axis in alphabetical order

Figure 6F. PLIN3 in *psd1*Δ *psd2*Δ

Tukey's multiple comparisons test		Adjusted P Value
A-B	0h vs. 6h	>0.9999
A-C	0h vs. 24h	>0.9999
A-D	0h vs. 0h	0.9994
A-E	0h vs. 6h	0.9727
A-F	0h vs. 24h	0.0017
A-G	0h vs. 0h	0.9957
A-H	0h vs. 6h	>0.9999
A-I	0h vs. 24h	>0.9999
A-J	0h vs. 0h	>0.9999
A-K	0h vs. 6h	>0.9999
A-L	0h vs. 24h	0.9985

Figure 6F. PLIN3 in *psd1*Δ *psd2*Δ (continued)

Tukey's multiple comparisons test		Adjusted P Value
B-C	6h vs. 24h	>0.9999
B-D	6h vs. 0h	0.9994
B-E	6h vs. 6h	0.9737
B-F	6h vs. 24h	0.0017
B-G	6h vs. 0h	0.9959
B-H	6h vs. 6h	>0.9999
B-I	6h vs. 24h	>0.9999
B-J	6h vs. 0h	>0.9999
B-K	6h vs. 6h	>0.9999
B-L	6h vs. 24h	0.9984
C-D	24h vs. 0h	0.9841
C-E	24h vs. 6h	0.9985
C-F	24h vs. 24h	0.0057
C-G	24h vs. 0h	>0.9999
C-H	24h vs. 6h	>0.9999
C-I	24h vs. 24h	>0.9999
C-J	24h vs. 0h	>0.9999
C-K	24h vs. 6h	>0.9999
C-L	24h vs. 24h	0.9724
D-E	0h vs. 6h	0.5918
D-F	0h vs. 24h	0.0001
D-G	0h vs. 0h	0.7673
D-H	0h vs. 6h	0.9297
D-I	0h vs. 24h	0.9986
D-J	0h vs. 0h	0.9932
D-K	0h vs. 6h	0.9994
D-L	0h vs. 24h	>0.9999
E-F	6h vs. 24h	0.0734
E-G	6h vs. 0h	>0.9999
E-H	6h vs. 6h	>0.9999
E-I	6h vs. 24h	0.9835
E-J	6h vs. 0h	0.9955
E-K	6h vs. 6h	0.9743
E-L	6h vs. 24h	0.5285
F-G	24h vs. 0h	0.0366
F-H	24h vs. 6h	0.0134
F-I	24h vs. 24h	0.0022
F-J	24h vs. 0h	0.0039
F-K	24h vs. 6h	0.0018
F-L	24h vs. 24h	<0.0001
G-H	0h vs. 6h	>0.9999
G-I	0h vs. 24h	0.9979
G-J	0h vs. 0h	0.9997
G-K	0h vs. 6h	0.9960
G-L	0h vs. 24h	0.7103
H-I	6h vs. 24h	>0.9999
H-J	6h vs. 0h	>0.9999
H-K	6h vs. 6h	>0.9999
H-L	6h vs. 24h	0.8983
I-J	24h vs. 0h	>0.9999
I-K	24h vs. 6h	>0.9999
I-L	24h vs. 24h	0.9967
J-K	0h vs. 6h	>0.9999
J-L	0h vs. 24h	0.9870
K-L	6h vs. 24h	0.9983

Letters indicate data groups as arranged on the x-axis in alphabetical order

Figure 7D. Short chain phospholipid

Unpaired t-test	P Value
PLIN1 - Cterm	0.0056
PLIN2 - 4helix	0.0015
PLIN5 - 4helix	0.0041

Figure 7E. ALDs

Unpaired t-test	P Value
PLIN1 - Cterm	0.0114
PLIN2 - 4helix	0.0020
PLIN4 - 4helix	0.0173
PLIN5 - 4helix	0.0034

Figure S3A. LD number per cell

Dunn's multiple comparisons test		Adjusted P Value
A-C	0h vs. 24h	>0.9999
C-F	24h vs. 24h	>0.9999
C-I	24h vs. 24h	>0.9999
D-F	0h vs. 24h	>0.9999
G-I	0h vs. 24h	0.1536
I-O	24h vs. 24h	>0.9999
J-L	0h vs. 24h	>0.9999
L-R	24h vs. 24h	0.9592
M-O	0h vs. 24h	>0.9999
P-R	0h vs. 24h	0.0498

Letters indicate data groups as arranged on the x-axis in alphabetical order

Figure S3B. LD area

Dunn's multiple comparisons test		Adjusted P Value
A-C	0h vs. 24h	<0.0001
D-F	0h vs. 24h	<0.0001
G-I	0h vs. 24h	<0.0001
J-L	0h vs. 24h	<0.0001
M-O	0h vs. 24h	<0.0001
P-R	0h vs. 24h	<0.0001
C-F	24h vs. 24h	0.1904
C-I	24h vs. 24h	0.0158
I-O	24h vs. 24h	>0.9999
L-R	24h vs. 24h	0.0418

Letters indicate data groups as arranged on the x-axis in alphabetical order

Figure S3D. Cycloheximide chase - WT + PLIN3

Tukey's multiple comparisons test	Adjusted P Value	
	-CHX	+CHX
0 vs. 1	0.8522	0.9987
0 vs. 2	0.9863	>0.9999
0 vs. 4	0.9959	0.9936
0 vs. 6	0.9815	0.9998
0 vs. 8	0.9967	0.9717
0 vs. 24	0.9928	0.0562
1 vs. 2	0.9983	>0.9999
1 vs. 4	0.4988	>0.9999
1 vs. 6	0.3797	>0.9999
1 vs. 8	0.5125	0.9997
1 vs. 24	0.4572	0.1579
2 vs. 4	0.8090	0.9995
2 vs. 6	0.6950	>0.9999
2 vs. 8	0.8202	0.9945
2 vs. 24	0.7727	0.0944
4 vs. 6	>0.9999	>0.9999
4 vs. 8	>0.9999	>0.9999
4 vs. 24	>0.9999	0.2135
6 vs. 8	>0.9999	0.9984
6 vs. 24	>0.9999	0.1224
8 vs. 24	>0.9999	0.3105

Figure S3F. Cycloheximide chase - WT + PLIN3-VL

Tukey's multiple comparisons test	Adjusted P Value	
	-CHX	+CHX
0 vs. 1	0.6055	0.9982
0 vs. 2	0.6255	0.9984
0 vs. 4	0.9272	>0.9999
0 vs. 6	>0.9999	>0.9999
0 vs. 8	0.9996	0.8499
0 vs. 24	0.9922	0.4318
1 vs. 2	>0.9999	>0.9999
1 vs. 4	0.9947	>0.9999
1 vs. 6	0.4586	>0.9999
1 vs. 8	0.3665	0.9864
1 vs. 24	0.2301	0.7523
2 vs. 4	0.9960	>0.9999
2 vs. 6	0.4778	>0.9999
2 vs. 8	0.3840	0.9853
2 vs. 24	0.2431	0.7454
4 vs. 6	0.8356	>0.9999
4 vs. 8	0.7513	0.9476
4 vs. 24	0.5752	0.6054
6 vs. 8	>0.9999	0.9334
6 vs. 24	0.9992	0.5710
8 vs. 24	>0.9999	0.9904

Figure S3H. Cycloheximide chase - *psd1Δ psd2Δ* + PLIN3

Tukey's multiple comparisons test	Adjusted P Value	
	-CHX	+CHX
0 vs. 1	0.6169	0.8918
0 vs. 2	0.6589	>0.9999
0 vs. 4	0.9939	>0.9999
0 vs. 6	0.9999	0.9989
0 vs. 8	>0.9999	0.9374
0 vs. 24	>0.9999	0.0053
1 vs. 2	>0.9999	0.9697
1 vs. 4	0.9371	0.9442
1 vs. 6	0.4210	0.6391
1 vs. 8	0.6913	>0.9999
1 vs. 24	0.5241	0.0889
2 vs. 4	0.9536	>0.9999
2 vs. 6	0.4606	0.9858
2 vs. 8	0.7313	0.9872
2 vs. 24	0.5662	0.0111
4 vs. 6	0.9550	0.9943
4 vs. 8	0.9980	0.9724
4 vs. 24	0.9825	0.0081
6 vs. 8	0.9993	0.7249
6 vs. 24	>0.9999	0.0015
8 vs. 24	>0.9999	0.0657

Figure S3J. Cycloheximide chase - *psd1Δ psd2Δ* + PLIN3-VL

Tukey's multiple comparisons test	Adjusted P Value	
	-CHX	+CHX
0 vs. 1	0.9423	>0.9999
0 vs. 2	0.8231	>0.9999
0 vs. 4	0.8952	0.9982
0 vs. 6	0.9562	0.9914
0 vs. 8	0.9251	0.9997
0 vs. 24	0.9635	>0.9999
1 vs. 2	0.9999	>0.9999
1 vs. 4	>0.9999	0.9987
1 vs. 6	>0.9999	0.9930
1 vs. 8	>0.9999	0.9998
1 vs. 24	>0.9999	>0.9999
2 vs. 4	>0.9999	0.9955
2 vs. 6	0.9997	0.9831
2 vs. 8	>0.9999	0.9989
2 vs. 24	0.9995	>0.9999
4 vs. 6	>0.9999	>0.9999
4 vs. 8	>0.9999	>0.9999
4 vs. 24	>0.9999	0.9990
6 vs. 8	>0.9999	>0.9999
6 vs. 24	>0.9999	0.9942
8 vs. 24	>0.9999	0.9999
